# Psychiatric symptoms influence reward‐seeking and loss‐avoidance decision‐making through common and distinct computational processes

**DOI:** 10.1111/pcn.13279

**Published:** 2021-07-17

**Authors:** Shinsuke Suzuki, Yuichi Yamashita, Kentaro Katahira

**Affiliations:** ^1^ Brain, Mind and Markets Laboratory, Department of Finance, Faculty of Business and Economics The University of Melbourne Melbourne Victoria Australia; ^2^ Frontier Research Institute for Interdisciplinary Sciences Tohoku University Sendai Japan; ^3^ Department of Information Medicine National Institute of Neuroscience, National Center of Neurology and Psychiatry Tokyo Japan; ^4^ Department of Psychological and Cognitive Sciences, Graduate School of Informatics Nagoya University Nagoya Japan; ^5^ Mental and Physical Functions Modeling Group, Human Informatics and Interaction Research Institute National Institute of Advanced Industrial Science and Technology (AIST) Tsukuba Japan

**Keywords:** computational psychiatry, decision‐making, loss, reinforcement learning, reward

## Abstract

**Aim:**

Psychiatric symptoms are often accompanied by impairments in decision‐making to attain rewards and avoid losses. However, due to the complex nature of mental disorders (e.g., high comorbidity), symptoms that are specifically associated with deficits in decision‐making remain unidentified. Furthermore, the influence of psychiatric symptoms on computations underpinning reward‐seeking and loss‐avoidance decision‐making remains elusive. Here, we aim to address these issues by leveraging a large‐scale online experiment and computational modeling.

**Methods:**

In the online experiment, we recruited 1900 non‐diagnostic participants from the general population. They performed either a reward‐seeking or loss‐avoidance decision‐making task, and subsequently completed questionnaires about psychiatric symptoms.

**Results:**

We found that one trans‐diagnostic dimension of psychiatric symptoms related to compulsive behavior and intrusive thought (CIT) was negatively correlated with overall decision‐making performance in both the reward‐seeking and loss‐avoidance tasks. A deeper analysis further revealed that, in both tasks, the CIT psychiatric dimension was associated with lower preference for the options that recently led to better outcomes (i.e. reward or no‐loss). On the other hand, in the reward‐seeking task only, the CIT dimension was associated with lower preference for recently unchosen options.

**Conclusion:**

These findings suggest that psychiatric symptoms influence the two types of decision‐making, reward‐seeking and loss‐avoidance, through both common and distinct computational processes.

Decision‐making to attain rewards and avoid losses is crucial for survival.^1^ Examples include the selection of hunting locations (e.g., forest or lake), routes of escape from predators, and targets of investment (e.g. risky equities or safe bonds). Recent studies in psychiatry have suggested that impairments in decision‐making are often accompanied by mental disorders, such as obsessive–compulsive disorder,^2^, ^3^ depression,^4^, ^5^ anxiety,^6^ and schizophrenia.^7^, ^8^, ^9^

Despite accumulating psychiatric evidence, the symptoms specifically coupled to impairments in decision‐making remain unclear. This obscurity is at least partly because of the complex nature of mental disorders (i.e. high comorbidity: coexistence of trans‐diagnostic symptoms).^10^, ^11^, ^12^ For example, half the number of individuals with a confirmed diagnosis of one mental disorder concurrently have additional disorders.^13^ In other words, psychiatric symptoms are likely to be interconnected beyond the diagnostic categories of mental disorders (e.g. obsessive–compulsive disorder, depression, and anxiety). This high interconnection makes it challenging and hinders the examination of the relationships between particular psychiatric symptoms and decision‐making.

To disentangle the interrelations of mental disorders, researchers have combined online experiments using general samples with a dimensional approach that aims to uncover the trans‐diagnostic dimensions of psychiatric symptoms.^14^, ^15^, ^16^ Online experiments are emerging as a powerful tool to efficiently collect large‐scale data in psychiatry.^17^ The data quality of online experiments is relatively good.^18^ For example, the test–retest reliability of self‐reported depression symptoms in online experiments was reported to be high.^18^ Furthermore, established findings in psychiatry and decision sciences could be properly replicated online.^14^

The dimensional view posits that the psychiatric state of each individual is characterized by a high dimensional space of behavioral symptoms (rather than a categorical ‘type’, such as depression, anxiety, and schizophrenia).^19^, ^20^ The dimensional view is compatible with large‐scale online experiments using general samples that do not treat clinical populations as separate groups. Analyses of large‐scale data based on the dimensional view would enable the identification of the hidden dimensions of psychiatric symptoms and the examination of their specific relationships with decision‐making.

How do psychiatric symptoms affect the decision‐making process? A large body of research in decision sciences has extensively described the decision‐making process using a machine learning algorithm known as reinforcement learning (RL).^21^ RL proposes that choices that recently led to reward delivery should be reinforced to improve future decisions. RL accounts for animal and human behavior as well as the underlying neural substrates of decision‐making to attain rewards.^22^, ^23^, ^24^ By utilizing RL and other computational frameworks, an emerging research field, known as computational psychiatry, has attempted to define the types of computations underlying decision‐making that are associated with psychiatric symptoms.^25^, ^26^, ^27^, ^28^, ^29^, ^30^

Nevertheless, the relationship between psychiatric symptoms and computations involved in decision‐making in two different contexts, reward‐seeking and loss‐avoidance, remains elusive. In decision neuroscience, a highly debated issue is whether common or distinct neurocomputational mechanisms mediate reward‐seeking and loss‐avoidance decision‐making.^31^, ^32^, ^33^ Several studies have addressed this issue in patients with major depression,^5^, ^34^, ^35^ obsessive–compulsive disorder,^36^, ^37^ mood and anxiety disorders,^6^ and schizophrenia.^8^, ^9^ For instance, schizophrenia and major depression have been reported to be coupled with low performance of reward‐seeking and loss‐avoidance decision‐making, as well as lower sensitivity to reward/loss feedback (lower learning rate and/or higher choice stochasticity in RL).^9^, ^35^ These studies, however, did not consider the inter‐connectivity of psychiatric symptoms (i.e. coexistence of trans‐diagnostic symptoms). To date, no studies in computational psychiatry have assessed the relationship between these two decision‐making contexts and psychiatric symptoms using a transdiagnostic approach.

To address this knowledge gap, the present study aimed to examine whether and how trans‐diagnostic dimensions of psychiatric symptoms are associated with reward‐seeking and loss‐avoidance decision‐making by leveraging a large‐scale online experiment combined with quantitative data analyses based on an RL framework.

## Methods

The study was approved by the ethics committee of the Department of Psychology, Graduate School of Informatics, Nagoya University (ID: NUPSY‐171027‐K‐01).

### Participants

In total, 2400 participants (1200 females; age range, 20–75 years; mean age ± SD, 47.94 ± 11.62 years) completed an online experiment consisting of a decision‐making task and various questionnaires. Participants were pre‐assessed to exclude those with a previous history of diagnosis of neurological/psychiatric illness based on a self‐report. To ensure data quality, in accordance with previous studies using online experiments,^14^, ^38^ 500 participants were excluded following careful assessments (see Methods S1). Data from the remaining 1900 participants (1000 females; age range, 20–69 years; mean age ± SD, 47.77 ± 11.62 years) were used in the data analyses. The exclusion rate (20.8%) was comparable to that in previous studies using online experimental platforms,^39^ and no particular exclusion criteria was applied to round the number of participants. Further details are provided in Methods S1.

### Decision‐making tasks

**Fig. 1:**
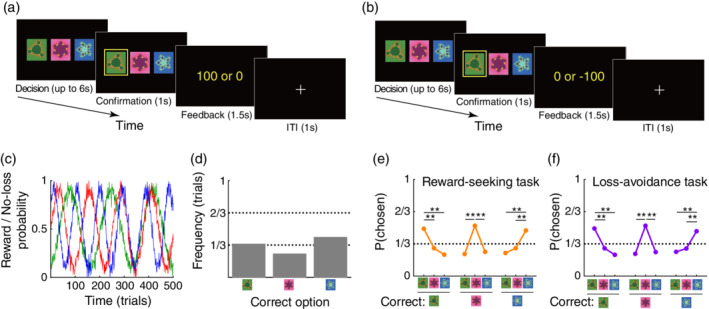
Experimental tasks. (a) Illustration of the reward‐seeking task. On each trial, participants selected one of the three options and either received a reward (100 JPY ~ 1 USD) or no‐reward (0 JPY) depending on the probability assigned to the chosen option. JPY, Japanese *Yen*. (b) Illustration of the loss‐avoidance task. On each trial, participants received a loss (−100 JPY) or no‐loss (0 JPY). (c) Reward and no‐loss probability for each option. The reward and no‐loss probabilities for the available options were identical between the two tasks. (d) Frequency of trials in which each of the three options had the highest reward or no‐loss probability (i.e. the correct option). (e) Proportion of each option chosen in the reward‐seeking task. Orange points and the error bars denote the mean and SEM across participants (*n* = 939). Note that the error bars overlap with the points. *Left*, the proportions when the green option had the highest reward probability (i.e. the correct option); *middle*, when the red option was the correct; and *right*, when the blue option was the correct. **P < 0.01; FDR‐corrected by the number of tests (i.e. 6) in a two‐tailed *t*‐test (corrected and uncorrected *Ps* < 0.001 for all the comparisons). (f) Proportion of each option chosen in the loss‐avoidance task (*n* = 961). Same format as in (e) (corrected and uncorrected *Ps* < 0.001 for all the comparisons).

In total, 939 participants performed a reward‐seeking task (Fig. 1a), and the remaining 961 participants performed a loss‐avoidance task (Fig. 1b). Note that they did not choose the task. Indeed, individuals who participated in October 2018 were assigned the reward‐seeking task, and those who participated in November and December 2018 were assigned the loss‐avoidance task. The demographic characteristics of the two groups were matched (Table S1). See Methods S1 for the details of reward payment.

In the reward‐seeking task, participants selected one of three options repeatedly (Fig. 1a), for a total of 500 trials, with a 1‐min break every 100 trials. On each of the 500 trials, participants received a reward (100 Japanese *yen* or 0 *yen*) depending on the probability assigned to the chosen option (see Methods S1 for a single trial's timeline). As the reward probability for each option was unknown to the participants and changed dynamically across trials (Fig. 1c), participants were required to keep track of the probabilities over the course of the task to maximize their reward earnings. Before initiating the experiment, the participants were informed that the reward probabilities may change during the task (no other information about the reward probabilities was provided). As all of the participants were confronted with the same reward probabilities, we were able to exclude the possibility that differences in the changing pattern of reward probabilities accounted for any individual differences in the participants' behavior. During this task, failure to respond was observed in 0.87 ± 1.75% of trials (mean ± SD).

In the loss‐avoidance task, participants received a loss (−100 Japanese *yen* or 0 *yen*) on each trial depending on the probability assigned to the chosen option (Fig. 1b). During this task, failure to respond was observed in 0.91 ± 1.79% of trials (mean ± SD). Notably, the no‐loss probabilities for the available options were identical to the reward probabilities in the reward‐seeking task (Fig. 1c).

### Questionnaires

After the decision‐making task, the participants were administered the Japanese versions of the following questionnaires: Schizotypal Personality Questionnaire Brief,^40^, ^41^ Obsessive–Compulsive Inventory,^42^ Self‐Rating Depression Scale,^43^, ^44^ State–Trait Anxiety Inventory,^45^, ^46^ and Barratt Impulsivity Scale.^47^, ^48^ Further details are presented in Methods S1 (Table S2).

### Factor analysis of questionnaire data

**Fig. 2:**
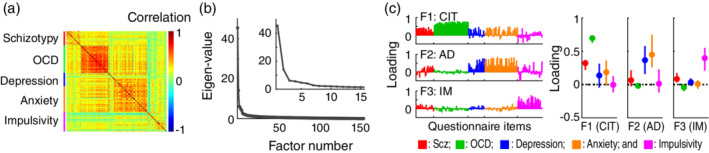
Factor analysis of questionnaire data. (a) Cross‐correlation of the responses to the individual 154 questionnaire items. Red, questionnaire items related to schizotypy (Scz); green, items related to obsessive–compulsive disorder (OCD); blue, items related to depression; orange, items related to anxiety; magenta, items related to impulsivity. (b) Scree plot. Dark gray points and lines denote eigenvalues derived from the cross‐correlation matrix in (a). The inset shows the same data focussing on factors 1 to 15. (c) Loadings in the three factors (dimensions) underlying psychiatric symptoms. *Left*, loadings of the 154 questionnaire items. *Right*, median, 25% quantile and 75% quantile of loadings within each questionnaire. CIT, compulsive behavior and intrusive thought; AD, anxiety‐depression; and IM, impulsivity.

To identify the trans‐diagnostic dimensions underlying psychiatric symptoms, we conducted a factor analysis (Fig. 2)^49^ on the participants' responses to 154 individual items in the five questionnaires. As responses to the items are categorical, the factor analysis was performed on a polychoric correlation matrix (Fig. 2a). The number of factors (i.e. 3) was determined based on Cattell's criterion,^50^ according to previous studies.^14^, ^15^ Specifically, we employed an objective implementation of this criterion, the Cattell‐Nelson‐Gorsuch (CNG) test, which compares the slope of all possible sets of three adjacent eigenvalues in the scree plot (Fig. 2b). Given the number of factors, the factor loadings were estimated by an *R* function (version 3.6.3), *fa*.^51^ Oblique rotation (‘Promax’) was applied, and the factor scores of each participant were estimated using the Harman algorithm. The three obtained factors corresponded to the trans‐diagnostic psychiatric dimensions. We labeled the three factors as ‘compulsive behaviour and intrusive thought (CIT)’, ‘anxiety‐depression (AD)’ and ‘impulsivity (IM)’, respectively (see RESULTS for the rationale).

Here, it is worth noting that the explained variances of the three factors were 0.18, 0.13 and 0.04 respectively; this implies that those factors explained only 35% of the variability in the questionnaire data. This is because the aim of the factor analysis in this study was to identify a small number of factors underlying various psychiatric symptoms, as in the previous computational psychiatry studies.^14^, ^15^ The explained variance would improve with the use of other criteria to determine the number of factors (e.g. parallel analysis).

### Psychiatric dimensions and overall task performance

**Fig. 3:**
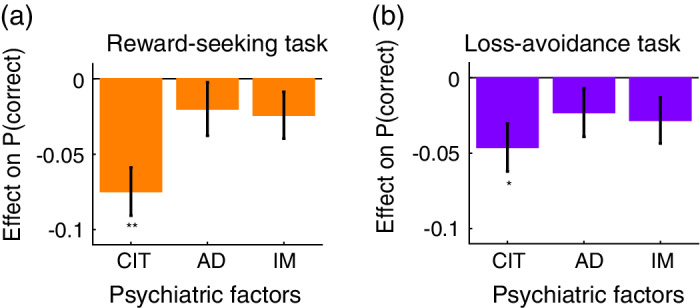
Psychiatric factors (dimensions) and decision‐making performance. (a) Effects of psychiatric factors on the proportion of correct choices in the reward‐seeking task (mean ± SEM, *n* = 465 435). A correct choice was defined as the selection of the option with the highest reward (or no‐loss) probability in a given trial. The mean and SEM of the effects were estimated with a generalized linear mixed‐effect model. **P < 0.01 and *P < 0.05, FDR‐corrected by the number of tests (i.e. 3) in two‐tailed *t*‐tests (effect of CIT: corrected and uncorrected *Ps* < 0.001; AD: corrected and uncorrected *Ps* = 0.255; and IM: corrected *P* = 0.179 and uncorrected *P* = 0.119). CIT, ‘compulsive behaviour and intrusive thought’; AD, ‘anxious‐depression’; and IM, ‘impulsivity’. (b) Effects of psychiatric factors on the proportion of correct choices in the loss‐avoidance task (mean ± SEM, *n* = 476 115). Same format as in (a) (effect of CIT: corrected *P* = 0.011 and uncorrected *P* = 0.004; AD: corrected and uncorrected *Ps* = 0.150; and IM: corrected *P* = 0.095 and uncorrected *P* = 0.064).

To examine the relationship between psychiatric factors (dimensions) and overall task performance (proportion of correct choices), we conducted a generalized linear mixed model (GLMM) analysis in each of the reward‐seeking and loss‐avoidance tasks: logit P(*correct*) ~ 1 + *CIT* + *AD* + *IM* + *age* + *sex* + *education* + *ses* + (1 | *participant*), with the model being specified by Wilkinson notation. Here, the correct choice was defined as that which provided the highest reward (or no‐loss) probability in a given trial. CIT, AD, and IM denote the *three* psychiatric factors. We also incorporated the participants' age, sex (coded as male: 1; female: 2), education‐level (coded as a junior high school diploma: 1; high school diploma: 2; technical school diploma: 3; vocational school diploma: 4; associate degree [community college diploma]: 5; bachelor's degree: 6; and a master's or doctorate degree: 7), and socioeconomic status (ses)^52^ as variables of no‐interest. All variables were *z*‐normalized and fed into regression analyses. The statistical significance of the regression coefficients was tested with a false discovery rate (FDR) multiple‐comparisons correction^53^ by the number of explanatory variables of interest (i.e. 3; see Fig. 3).

To compare CIT effects between tasks, we developed another GLMM analyzing the data from the two tasks together and including the interaction term of CIT and the task: logit P(*correct*) ~ 1 + (*CIT* + *AD* + *IM* + *age* + *sex* + *education* + ses) * task + (1 | *participant*), where the ‘task’ coded the reward‐seeking task as 1 and the loss‐avoidance task as −1.

### Psychiatric dimensions and decision‐making processes: Mixed‐effect regression analysis

To examine how past rewards (no‐losses) and choices affected the participants' current behavior, and how these effects were modulated by psychiatric factors (dimensions), we conducted a GLMM analysis. A full description of the GLMM is provided in Methods S2.

**Fig. 4:**
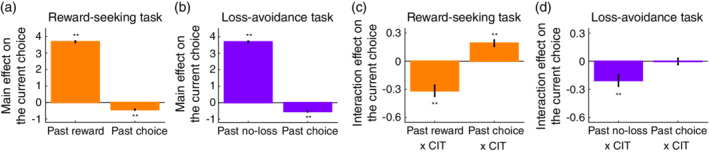
Psychiatric factors (dimensions) and decision‐making processes. (a) Main effects of past rewards and past choices on the current choice in the reward‐seeking task (mean ± SEM, *n* = 461 679). The mean and SEM of the effects were estimated with a generalized linear mixed‐effect model (GLMM). **P < 0.01, FDR‐corrected by the number of tests (i.e. 2) in two‐tailed *t*‐test (Past reward: corrected and uncorrected *Ps* < 0.001; Past choice: corrected and uncorrected *Ps* < 0.001). (b) Main effects of past no‐losses and past choices on the current choice in the loss‐avoidance task (mean ± SEM, *n* = 472 271). Same format as in (a) (Past no‐loss: corrected and uncorrected *Ps* < 0.001; Past choice: corrected and uncorrected *Ps* < 0.001). (c) Interaction effects on the current choice in the reward‐seeking task (mean ± SEM, *n* = 461 679). The mean and SEM of the interaction effects were estimated with the same GLMM as in (a). *Left*, interaction effect between past reward and CIT; and *right*, interaction effect between past choice and CIT. **P < 0.01, FDR‐corrected by the number of tests (i.e. 2) in two‐tailed *t*‐tests (Past reward x CIT: corrected and uncorrected *Ps* < 0.001; Past choice x CIT: corrected and uncorrected *Ps* < 0.001). CIT, compulsive behavior and intrusive thought. (d) Interaction effects on the current choice in the loss‐avoidance task (mean ± SEM, *n* = 472 271). Same format as in (c) (Past no‐loss x CIT: corrected and uncorrected *Ps* < 0.001; Past choice x CIT: corrected and uncorrected *Ps* = 0.934).

In the reward‐seeking task, the GLMM was defined as follows (in the Wilkinson notation): logit P(*C*_*t*_) ~ 1 + (*R*_*t*‐1_ + *R*_*t*‐2_ + *R*_*t*‐3_ + *R*_*t*‐4_ + *C*_*t*‐1_ + *C*_*t*‐2_ + *C*_*t*‐3_ + *C*_*t*‐4_) * (*CIT* + *AD* + *IM* + *age* + *sex* + *education* + *ses*) + (1 + *R*_*t*‐1_ + *R*_*t*‐2_ + *R*_*t*‐3_ + *R*_*t*‐4_ + *C*_*t*‐1_ + *C*_*t*‐2_ + *C*_*t*‐3_ + *C*_*t*‐4_ | *participant*), where $R_{t-\tau }$ and $C_{t-\tau }$ denote recent past rewards and recent past choices, respectively (trials *t* − 1, *t* − 2, *t* − 3, and *t* − 4). Here, $R_{t-\tau }$ was coded as 1 if the participant chose *X* and obtained a reward on trial t−τ, −1 if they chose *Y* or *Z* and obtained a reward, and 0 if there was no reward. $C_{t-\tau }$ was coded as 1 if the participant chose *X* on trial t−τ, and − 1 otherwise. The total main effect of past rewards over the past *four* trials can be derived from $\beta \left(R\right)=\sum_{i=1}^{4}\beta \left(R_{t-i}\right)$, where $\beta \left(R_{t-i}\right)$ denotes the regression coefficient of the variable $R_{t-i}$. The total main effect of past choices as well as the total interaction effects with psychiatric factors can be derived in the same way. We tested the statistical significance of the effects with an FDR multiple‐comparison correction (Fig. 4). In the loss‐avoidance task, the same GLMM was employed, treating ‘no‐loss’ and ‘loss’ as ‘reward’ and ‘no‐reward’, respectively.

To directly compare the effects between tasks, we developed another GLMM to analyze the data from the two tasks together and included the interaction terms with the task (1 for reward‐seeking and −1 for loss‐avoidance task): logit P(*C*_*t*_) ~ 1 + (*R*_*t*‐1_ + *R*_*t*‐2_ + *R*_*t*‐3_ + *R*_*t*‐4_ + *C*_*t*‐1_ + *C*_*t*‐2_ + *C*_*t*‐3_ + *C*_*t*‐4_) * (*CIT* + *AD* + *IM* + *age* + *sex* + *education* + *ses*) * task + (1 + *R*_*t*‐1_ + *R*_*t*‐2_ + *R*_*t*‐3_ + *R*_*t*‐4_ + *C*_*t*‐1_ + *C*_*t*‐2_ + *C*_*t*‐3_ + *C*_*t*‐4_ | *participant*).

### Psychiatric dimensions and decision‐making processes: A model‐based analysis

We constructed computational models and fitted them to the participants' choice behaviors in the decision‐making tasks. Details are provided in Methods S2.

**RL1a.** The first model was a conventional RL model, termed Q‐learning.^21^ In this model, an agent makes a choice on each trial depending on the value of each option. The choice probability of each option is given by the *Q* values of the options (i.e. Softmax function). Here, the parameter $\beta \;\in \;\left[0\right.,\left.\infty \right)$ governs the degree of stochasticity in the choices (termed *inverse‐temperature*).^21^ Once a choice is made and the reward outcome is revealed, the agent updates the value of the chosen option based on the reward prediction error with a learning rate $\alpha \;\in \;\left[0,1\right].$ ^21^

**RL1b.** This model is almost identical to RL1a but includes ‘value‐forgetting’.^54^, ^55^, ^56^, ^57^ That is, the values of the unchosen options are forgotten (i.e. decay with time). In other words, on each trial, an agent updates not only the value of the chosen option but also the values of the unchosen options.

**RL2a.** In this model, an agent considers values as well as choice‐traces when making a decision.^58^, ^59^, ^60^ The choice‐trace of each option functions in decision‐making with the parameter $\gamma \;\in \;\left(-\infty ,\infty \right)$, which denotes the *weight of the choice‐traces*. Individuals with positive choice‐trace weights are likely to repeat the recently selected choice. Conversely, individuals with negative choice‐trace weights tend to avoid a recently selected option.

**RL2b.** This model is almost identical to RL2a but includes value‐forgetting as in RL1b.

**RL3a.** This model has differential learning rates for positive and negative reward prediction errors. The process of decision‐making is identical to that of RL1a.

**RL3b.** This model is almost identical to RL3a but includes value‐forgetting.

**RL4a.** This model is an empirically well‐supported Pearce‐Hall model,^61^ where the learning rate can be adaptively modulated. In the decision‐making tasks with dynamically changing reward (no‐loss) probability, this type of model would provide a good fit.

**RL4b.** This model is almost identical to RL4a but includes value‐forgetting.

Given the negative coupling between the psychiatric factor CIT and the decision‐making performance (see RESULTS), we carried out a linear regression analysis to examine the relationship between the psychiatric factor CIT and three parameters in the best‐fitted model (i.e. the learning rate *α*, inverse temperature *β*, and choice‐trace weight *γ* in RL2b; see RESULTS for the motivation to focus on these parameters): *CIT* ~ 1 + *α* + *β* + *γ* + *age* + *sex* + *education* + *ses* + *α*_*F*_ + *α*_*C*_ + *AD* + *IM*. Note that *β* was log‐transformed due to the severe non‐normality (skewness >2 and kurtosis >7). To control potential confounding effects, the regression model includes various variables of no interest: age, sex, education level, socioeconomic status (ses), psychiatric factors (AD and IM), and the other parameters in RL2b (the forgetting rate *α*_*F*_ and the choice‐trace decay rate *α*_*C*_; see Methods S2 for details).

### Predictability of psychiatric dimensions on the decision‐making

To assess the predictive power of the psychiatric factors (CIT, AD and IM) on the task performance and the learning rate, we evaluated the prediction accuracy of linear regression models (*Y* ~ *CIT* + *AD* + *IM*). Here, *Y* denotes the predicted variable (i.e. task performance [proportion of correct choices] and learning rate). Furthermore, to evaluate the predictive power of the CIT factor, we also examined the extent to which the accuracy is reduced by omitting CIT from the prediction model (i.e. *Y* ~ *AD* + *IM*). In these analyses, prediction accuracies were assessed by the cross‐validated (leave‐one‐out) correlation between predicted and actual values.

## Results

### Experimental task and basic behavior

We confirmed that in both the decision‐making tasks, the participants were more likely to choose the correct option that had the highest reward (or no‐loss) probability in a given trial (*P* < 0.01; Fig. 1e,f) as compared to the other options. The data suggests that participants generally succeeded in learning the reward/no‐loss probabilities.

### Three dimensions underlying psychiatric symptoms: Factor analysis

Consistent with the comorbidity of mental disorders, we found high correlations between the total questionnaires' scores (Fig. S1a). For instance, scores of depression‐ and anxiety‐related questionnaires shared a large amount of the variance (r^2^ = 0.69). Furthermore, the shared variance between questionnaires related to depression and obsessive–compulsive disorder was 0.28. These high collinearities made it difficult to examine the relationship between individual questionnaire scores and decision‐making performance.

To disentangle this inter‐correlated structure, we sought to identify the dissociable trans‐diagnostic dimensions of multiple psychiatric symptoms by applying factor analysis to the questionnaire data. The factor analysis revealed three hidden factors (‘dimensions’) spanning 154 items from the five questionnaires (Fig. 2 and Table S3; see METHODS for details). The first factor (F1) had higher loadings from symptoms associated with obsessive–compulsive disorder (median loading = 0.70, 25% quantile = 0.63 and 75% quantile = 0.74) and schizotypy (median = 0.32, 25% quantile = 0.22 and 75% quantile = 0.37) (Fig. 2c), as well as moderate loadings from depression (median = 0.14, 25% quantile = −0.05 and 75% quantile = 0.31) and anxiety (median = 0.19, 25% quantile = −0.01 and 75% quantile = 0.36). The second factor (F2) had higher loadings from symptoms associated with depression (median = 0.37, 25% quantile = 0.16 and 75% quantile = 0.58) and anxiety (median = 0.45, 25% quantile = 0.26 and 75% quantile = 0.75) (Fig. 2c). According to previous studies,^14^, ^15^ we labeled the two factors as ‘compulsive behaviour and intrusive thought (CIT)’ and ‘anxious‐depression (AD)’ respectively. The third factor (F3) was labeled as ‘impulsivity (IM)’ as it is primarily coupled with impulsivity‐related symptoms (median loading = 0.40, 25% quantile = 0.21 and 75% quantile = 0.55) (Fig. 2c). Remarkably, these three factors shared at most only 15% variance (r^2^ = 0.14 between CIT and IM), allowing us to adequately examine the relationship between the psychiatric dimensions and decision‐making performance. In summary, the results of the factor analysis support the existence of multiple hidden dimensions underlying mutually related psychiatric symptoms.

### Psychiatric dimensions and overall decision‐making performance: Mixed‐effect regression analysis

GLMM analyses revealed that, in both reward‐seeking and loss‐avoidance tasks, the CIT factor (‘compulsive behaviour and intrusive thought’) had a negative impact on decision‐making performance (*Ps* < 0.05; Fig. 3a,b). CIT was negatively coupled with the proportion of correct choices (i.e. selecting the option with the highest reward/no‐loss probability), while controlling for age, sex, education level, and socio‐economic status of the participants. On the other hand, the AD (‘anxious‐depression’) and IM (‘impulsivity’) factors were not significantly associated with decision‐making performance (*Ps* > 0.09; Fig. 3a,b).

To directly compare the negative effects of CIT between the two decision‐making tasks, we then constructed another GLMM to analyze the data from the two tasks together including the interaction term of CIT and the task. This additional analysis did not show a significant interaction effect (*P* = 0.20; see Fig. S2), indicating null evidence for the differential impacts of CIT on the overall decision‐making performance between the two tasks.

Furthermore, we found little associations between total questionnaires' scores and decision‐making performance (Fig. S1b), possibly due to the high collinearities among scores (Fig. S1a). Regressing the total scores against the proportion of correct choices, GLMM analyses revealed only one significant association (i.e. the OCD‐related questionnaire and the performance in the reward‐seeking task; see Fig. S1b). The results do not change essentially if we analyzed the scores for state and trait anxiety separately (Fig. S1c,d).

### Psychiatric dimensions and decision‐making processes: Mixed‐effect regression analysis

We next questioned *how* the CIT (‘compulsive behaviour and intrusive thought’) factor, associated with underperformance in decision‐making, was related to specific computational processes in decision‐making. To achieve this objective, we assessed the participants' behavioral patterns in the decision‐making tasks and tested whether and how these behavioral patterns were modulated by CIT.

The RL account of decision‐making predicts that an individual's behavior is driven by reward feedback. Choices that lead to reward delivery or loss avoidance are reinforced and, therefore, are more likely to be selected in the future. In addition, studies have reported that the individuals' behavior is also guided by their past choices.^58^, ^59^, ^62^ To quantify these effects, we employed a GLMM to test the main effects of past rewards (or no‐losses) and past choices on the participants' behavior, and determine how these main effects were modulated by the CIT factor (i.e. interaction effects), while controlling for the potential confounding effects of age, sex, education level, and socio‐economic status as well as the remaining psychiatric factors (i.e. AD: ‘anxious‐depression’, and IM: ‘impulsivity’).

We first examined the main effects of reward and choice history on behavior independently of psychiatric factors. Consistent with RL account predictions, in both reward‐seeking and loss‐avoidance tasks, we found positive impacts of reward and no‐loss history (*P* < 0.01; Fig. 4a,b). Furthermore, the effect of choice history on behavior was significantly negative in both tasks (*P* < 0.01; Fig. 4a,b), suggesting that the participants preferred options that had not been recently chosen.

We next examined how the CIT factor modulated the effects of past rewards (no‐losses) and choices on current behavior (i.e. interaction effects). In both reward‐seeking and loss‐avoidance tasks, the positive effects of reward history were attenuated by CIT (Fig. 4c,d). Specifically, the regression coefficients of the interaction between reward history and CIT were significantly negative (*P* < 0.01; Fig. 4c,d).

On the other hand, the negative effects of choice history were modulated by the CIT factor only in the reward‐seeking task (Fig. 4c). The regression coefficient of the interaction term between choice history and CIT was positive in the reward‐seeking task (*P* < 0.01; Fig. 4c), indicating that the negative effect of choice history was attenuated by CIT. In other words, as CIT increased, participants tended to more stick to the previous choices. In the loss‐avoidance task, we observed a no significant interaction effect between choice history and CIT (*P* = 0.93; Fig. 4d).

Next, we directly compared the above interaction effects between the two decision‐making tasks. A new GLMM showed a significant effect of the three‐way interaction among choice history, CIT, and task (*P* < 0.01; see Fig. S3), indicating that the association between CIT and the effect of choice history was more prominent in the reward‐seeking task than in the loss‐avoidance task. However, the three‐way interaction among reward history, CIT and task was not significant (*P* = 0.19; see Fig. S3), implying no difference in the association between CIT and the effect of reward history between the two tasks.

Finally, we tested for the effects of history length. In the original analyses, we considered the reward and choice history of four previous trials (i.e. *t* ‐ 1,… *t* ‐ 4; see METHODS). Additional analysis confirmed that the original results were essentially unchanged within the range of 2–6 of history length (Fig. S3b–i).

### Psychiatric dimensions and decision‐making processes: Model‐based analysis

**Fig. 5:**
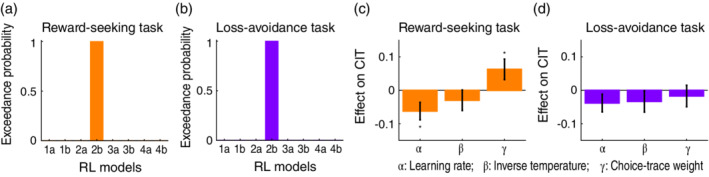
Computational model‐based analysis of the relationship between psychiatric factors (dimensions) and decision‐making processes. (a) Model comparison in the reward‐seeking task. Each bar denotes exceedance probability in Bayesian model selection^63^ (higher values close to one indicate a better fit). RL, reinforcement learning. (b) Model comparison in the loss‐avoidance task. Same format as in (a). (c) Effects of RL parameters on the CIT factor in the reward‐seeking task (mean ± SEM, *n* = 939). *P < 0.05, FDR‐corrected by the number of tests (i.e. 3) in one‐tailed *t*‐tests (α: corrected *P* = 0.026 and uncorrected *P* = 0.009; β: corrected and uncorrected *Ps* = 0.166; γ: corrected *P* = 0.031 and uncorrected *P* = 0.021). CIT, compulsive behavior and intrusive thought. (d) Effects of RL parameters on the CIT factor in the loss‐avoidance task (mean  ±  SEM, *n* = 961). Same format as in (c) (α: corrected *P* = 0.225 and uncorrected *P* = 0.078; β: corrected *P* = 0.225 and uncorrected *P* = 0.150; γ: corrected and uncorrected *Ps* = 0.300).

To develop further insights into computational processes, we fitted RL models to the participants' behavior and correlated the best‐fit model parameters with the CIT factor (‘compulsive behaviour and intrusive thought’) associated with underperformance in decision‐making. Consistent with the above GLMM analyses, a formal model comparison^63^ revealed that in both reward‐seeking and loss‐avoidance tasks, a model including choice‐trace effects (i.e. RL2b) provided a better fit than the alternative models (Fig. 5a,b; and see Fig. S4 and Methods S2 for the validation of the model fitting based on the simulation data). The best‐fitted model comprised three critical parameters: *learning rate*, which governs the degree to which the value of the chosen option is updated in proportion to the reward prediction error; *inverse‐temperature*, which governs the sensitivity of choices to option values (i.e. choice stochasticity); and *choice‐trace weight*, which determines the degree to which past choices affect the individual's current behavior.

Importantly, previous theoretical work from our group^64^ showed that the learning rate, inverse temperature, and choice‐trace weight can be reflected in the effects of past rewards (or no‐losses) and past choices. Specifically, the attenuated (positive) effects of past rewards by CIT (see Fig. 4) can reflect a lower learning rate and/or inverse temperature, while the attenuated (negative) effects of past choices (see Fig. 4a,c) can reflect higher choice‐trace weight and/or lower inverse temperature. Therefore, we hypothesized that, in both the reward‐seeking and loss‐avoidance tasks, learning rate and/or inverse temperature was *negatively* correlated with CIT; and that, only in the reward‐seeking task, choice‐trace weight and/or inverse temperature was positively and negatively correlated with CIT *respectively*. Consistent with the hypotheses, in the reward‐seeking task, a linear regression revealed that CIT was negatively coupled with the learning rate and positively with the choice‐trace weight (*Ps* < 0.05; Fig. 5c), while controlling for the effects of age, sex, education level, and socio‐economic status as well as the other RL parameters and psychiatric factors. In the loss‐avoidance task, we did not find robust associations between CIT and learning rate or inverse temperature (*Ps* > 0.14; Fig. 5d), while the learning rate was found to be significantly coupled with CIT (*P* < 0.01; see Fig. S5) without controlling for the effects of demographic information (i.e. age, sex, education level, and socio‐economic status).

### Additional analysis with reduced questionnaire data

To make interpretations easier during factor analysis, researchers sometimes ignore low‐loading items based on an arbitrary cut‐off value (e.g. 0.4). Following the practice, we discarded the questionnaire items with a loading of less than 0.4 (Fig. 2 and Table S3), and re‐analyzed the reduced data. The additional analyses essentially demonstrated no change from the original results (Figs 2, 3, 4, 5) on omission of the low‐loading items (Fig. S6).

### Predictability of psychiatric dimensions on the decision‐making

Finally, we assessed the predictability of psychiatric factors (CIT, AD and IM) on task performance and learning rate. Furthermore, to evaluate the contribution of CIT to the prediction, we assessed the extent to which the accuracy is reduced by omitting CIT from the prediction model. In the reward‐seeking task, the prediction accuracy of the psychiatric factors on the performance was found to be 0.12, which is significantly greater than that of a partial model without CIT (*P* < 0.001, *permutation* test with permuting the classification labels 10 000 times). In the loss‐avoidance task, the prediction accuracy of the three factors was 0.09, which is marginally greater than that of the partial model (*P* = 0.077, *permutation* test). With regard to the learning rate, the prediction accuracies of the three psychiatric factors were 0.12 and 0.07 in the reward‐seeking and the loss‐avoidance tasks, respectively. These accuracies were not significantly different from those of the partial models that omit CIT (*P* = 0.411 in the reward‐seeking task and *P* = 0.244 in the loss‐avoidance task, *permutation* test).

## Discussion

In this study, we examined the relationship between psychiatric symptoms and impairments in reward‐seeking and loss‐avoidance decision‐making by leveraging a large‐scale online experiment combined with a dimensional approach and quantitative analysis of behavior.

First, we identified *three* trans‐diagnostic dimensions of psychiatric symptoms, labeled ‘compulsive behaviour and intrusive thought (CIT)’, ‘anxiety‐depression (AD)’, and ‘impulsivity (IM)’. CIT had higher loadings from obsessive–compulsive and schizotypal symptoms as well as moderate loadings from depressive and anxious symptoms, while AD had higher loadings from depressive and anxious symptoms, and IM from impulsivity. The pattern of loadings was overall consistent with previous online studies^14^, ^15^ (except for IM) as well as a popular model in psychopathology that suggests the existence of the following three dimensions underlying mental disorders^10^, ^65^, ^66^, ^67^: *thought disorder* (e.g. obsessive–compulsive disorder and schizophrenia), *internalizing disorder* (e.g. major depression and anxiety disorder) and *externalizing disorder* (e.g. conduct disorder including problematic impulsivity).

The CIT dimension was found to be coupled with impairments in both reward‐seeking and loss‐avoidance decision‐making (Fig. 3). This finding is consistent with previous studies showing the impairments in reward‐seeking and loss‐avoidance decision‐making in people with obsessive–compulsive disorder^36^, ^37^ and schizophrenia.^8^, ^9^, ^68^ Of note, other studies have also reported deficits in the two types of decision‐making in depression^5^, ^34^, ^35^ and anxiety.^6^ These previous reports would also be consistent with our results, considering that the CIT dimension has moderate loadings from depressive and anxious symptoms (Fig. 2c). In other words, decision‐making deficits would be associated not only with obsessive–compulsive and schizotypal symptoms, but also with components of depressive and anxious symptoms contained in the CIT dimension (note: the symptoms of depression and anxiety highly associated with CIT did not substantially overlap with those associated with AD; see Fig. S7). Our findings extend results from past psychiatric studies by identifying a trans‐diagnostic dimension associated with impairments in reward‐seeking and loss‐avoidance decision‐making.

In both reward‐seeking and loss‐avoidance decision‐making, the CIT dimension was coupled with weaker effects of past rewards (no‐losses) on current behavior. Specifically, the positive effects of past rewards were attenuated by CIT (Fig. 4), suggesting that psychiatric symptoms related to CIT were associated with a lower preference for options that recently led to rewards or no‐losses. Theoretically, a lower sensitivity to past rewards (losses) may reflect *learning rate* or *inverse temperature* (i.e. choice stochasticity) in RL, or other complicated processes.^64^, ^69^ Practically, dissociating these possibilities by model fitting is, however, non‐trivial. Indeed, while insensitivity to rewards has long been considered to be a core symptom of mood disorders, the field of computational psychiatry has yet to reach a consensus on which of the two parameters (learning rate or inverse temperature) is primarily associated with this symptom.^34^, ^35^, ^70^, ^71^, ^72^, ^73^ In patients with schizophrenia, one study suggested the importance of inverse temperature in reward‐seeking and loss‐avoidance decision‐making.^9^ Our model‐based analysis suggests that the lower sensitivity associated with CIT resulted from a decrease in the learning rate despite the result in the loss‐avoidance task not being robust. One avenue for future research would be to tackle this issue by employing novel approaches (e.g. mapping participants' behavior into a low‐dimensional space by recurrent neural networks, and relating the dimensions to psychiatric symptoms^69^).

The CIT dimension attenuated the negative effects of past choices on the current behavior only in reward‐seeking decision‐making (Fig. 4), which suggests that psychiatric symptoms related to CIT were associated with lower preference for recently unchosen options. One interpretation of this result is that CIT was associated with a decline in motivation to explore unfamiliar options to resolve uncertainty, as the values of unchosen options are uncertain. Consistent with this interpretation, uncertainty‐driven exploratory behavior is reduced in patients with schizophrenia.^74^, ^75^ Another interpretation is that CIT was simply associated with an increase in the tendency to repeat the same choice (i.e. ‘choice perseverance’, ‘choice stickiness’, or ‘decision inertia’). This interpretation is largely coherent with a prevailing hypothesis on the OCD endophenotype suggesting that patients with OCD lose cognitive flexibility^76^, ^77^, ^78^ (but see references^79^, ^80^ for counter examples). Nevertheless, these two interpretations are not dissociable in conventional decision‐making tasks (e.g. multi‐armed bandit tasks). Therefore, more sophisticated experimental tasks^81^, ^82^ are required to dissect the distinct psychological mechanisms at play in this process.

As discussed above, the CIT dimension was associated with lower preference for choice options that recently led to rewards or no‐losses in both reward‐seeking and loss‐avoidance decision‐making, while being associated with lower preference for recently unchosen options only in loss‐avoidance decision‐making. The results broadly suggest that psychiatric symptoms, especially compulsive behavior and intrusive thought, influence the two types of decision‐making through common and distinct computational processes. Furthermore, the differential impact of CIT in the two types of decision‐making implies that our framing manipulation of reward‐seeking and loss‐avoidance contexts was effective (see METHODS). Moreover, the results have implications in the field of decision neuroscience. To date, whether common or distinct neurocomputational mechanisms underpin reward‐seeking and loss‐avoidance decision‐making remains elusive.^31^, ^32^, ^33^ Our findings imply that a common mechanism underlies outcome processing (e.g. reward and loss); however, distinct mechanisms underlie the processing of an individual's own choice.

It is worth noting that, in our data, the psychiatric dimensions were not very predictive of individual differences in decision‐making. The prediction accuracies (cross‐validated correlations between the predicted and actual values) were found to be 0.12 at best. More broadly, recent studies in computational psychiatry have demonstrated significant effects of psychiatric symptoms on decision‐making and learning using large‐scale online experiments on the general samples. Effect sizes, however, are quite small, meaning that hundreds of participants are required to reliably detect the effects.^14^, ^15^ An important direction of future research would be to conduct a large‐scale experiment on the clinical samples.

There are certain limitations to this study. We did not include participants with a history of diagnosis of neurological/psychiatric illness. This is because participation in the experiment (e.g. answering the questionnaires of psychiatric symptoms) may have a negative impact on the mental state, but we could not provide effective countermeasures (e.g. introducing to a psychiatrist) online. We therefore decided not to recruit people with a diagnosis, who are considered to be vulnerable to this adverse effect. While online experiments on the general samples have been considered a powerful tool in psychiatry,^17^ we should be cautious with regard to the extent that the present findings are applicable to clinical populations. Examination of their generalizability warrants a future study.

Another caveat is that, in our decision‐making tasks, all participants were confronted with the same sequence of reward and loss‐avoidance probabilities. This design was introduced to exclude the possibility that differences in the changing pattern of reward and loss‐avoidance probabilities accounted for any individual differences in the participants' behavior. In the future, testing whether the findings obtained in this study are replicated with other reward and loss‐avoidance probability sequences is needed. Furthermore, in our experiment, different groups of participants took part in the reward‐seeking and loss‐avoidance tasks (however, the demographic characteristics of the two groups were matched: see Table S1). We employed a between‐participant design to collect as much choice data as possible for each participant (i.e. 500 trials), which is of particular importance for compensating for the potential low‐quality of online data.

In conclusion, this study provides insights into the effects of psychiatric symptoms on human decision‐making in various contexts (i.e. reward‐seeking and loss‐avoidance). Our findings suggest that a trans‐diagnostic psychiatric dimension, ‘compulsive behaviour and intrusive thought’, influences reward‐seeking and loss‐avoidance decision‐making through common and distinct computational processes. We believe the present study makes an essential contribution to the unraveling of the complex relationship between psychiatric symptoms and psychological/computational processes involved in decision‐making.

## Disclosure statement

This work was supported by the JSPS KAKENHI Grants: 17H05933 and 17H06022 (S.S.), 17H06039, 19H04998, 20H00625 (Y.Y.), 17H05946, 18KT0021 (K.K.), and the JST CREST Grant JPMJCR16E2 (Y.Y.). The authors report no biomedical financial interests or potential conflicts of interest.

## Author contributions

S.S., Y.Y. and K.K. designed the research; S.S. performed the research; S.S. analyzed the data; and S.S., Y.Y. and K.K. wrote the paper.

## Supporting information

**Table S1** Demographic information of the participants who performed the reward‐seeking (*n* = 939) and loss‐avoidance (*n* = 961) tasks.
**Table S2** Total scores of the questionnaires (*n* = 1900).
**Table S3** Loadings of each item for the three factors (*n* = 1900).
**Method S2** Data Analysis.
**Figure S1** Supplementary analysis on the total scores of the questionnaires.
**Figure S2** Supplementary analysis of psychiatric factors (dimensions) and decision‐making performance.
**Figure S3** Supplementary analysis of psychiatric factors (dimensions) and decision‐making processes.
**Figure S4** Validation of computational models and model‐fitting procedure based on simulation data
**Figure S5** Supplementary computational model‐based analysis.
**Figure S6** Supplementary analysis using the reduced questionnaire data.
**Figure S7** Supplementary factor analysis of questionnaire data.
**Method S1** Experimental Design.
