# Supplementary material for: Psychiatric symptoms influence reward‐seeking and loss‐avoidance decision‐making through common and distinct computational processes
Source: Psychiatry Clin Neurosci. 2021 Jul 17;75(9):277–85. doi: 10.1111/pcn.13279 (PMC8457174; doi:10.1111/pcn.13279)
Supplement: Supplementary file 1 — Table S1 Demographic information of the participants who performed the reward‐seeking (n = 939) and loss‐avoidance (n = 961) tasks. Table S2 Total scores of the questionnaires (n = 1900). Table S3 Loadings of each item for the three factors (n = 1900). Method S2 Data Analysis. Figure S1 Supplementary analysis on the total scores of the questionnaires. Figure S2 Supplementary analysis of psychiatric factors (dimensions) and decision‐making performance. Figure S3 Supplementary analysis of psychiatric factors (dimensions) and decision‐making processes. Figure S4 Validation of computational models and model‐fitting procedure based on simulation data Figure S5 Supplementary computational model‐based analysis. Figure S6 Supplementary analysis using the reduced questionnaire data. Figure S7 Supplementary factor analysis of questionnaire data. Method S1 Experimental Design. [file PCN-75-277-s001.pdf]

## Method S1: Experimental Design

### Participants

A total of 2,400 participants (1,200 females; age range, 20–75 years; mean age ± SD, 47.94 ± 11.62 years) completed the online experiment consisting of a decision-making task and questionnaires. Participants provided responses in more than 90% of trials in the decision-making task and provided answers to all questionnaire items. Data were collected using an online research company, Cross Marketing Inc. (http://global.cross-m.co.jp/). The research company did not play any role in experimental design, data analysis, or writing of the manuscript. Participants were all native Japanese speakers and pre-assessed to exclude those with a previous history of diagnosis of neurological/psychiatric illness based on the self-report. All participants provided their informed consent online by clicking 'I Agree' after reading the instructions for the experiment. We did not use any statistical methods to predetermine the sample size. Our sample size selection was based on that used in previous studies [1].

To ensure data quality in the online experiment and agreement with previous studies [1,2], we excluded 500 participants after careful assessments. In total, 431 participants who were not serious about the questionnaire were excluded. To identify these participants, we included a catch item, "If you have carefully read the questions so far, please select 'a little' as your answer", in the questionnaire (see Questionnaires in METHODS). If participants failed to choose the appropriate response, they were excluded from subsequent analyses. Of the remaining participants, eight were excluded as they did not provide information about their education level. Finally, we excluded 61 participants who chose only one option on more than two-thirds of the trials in the reward-seeking or loss-avoidance decision-making tasks. Choices of one option over the course of trials (e.g., more than *two-thirds* of the trials) were highly irrelevant in terms of the task demands, given that the frequencies of trials in which each of the three options had the highest reward probability were almost the same (Fig. 1cd). The data from the remaining 1,900 participants (1,000 women; age range, 20–69 years; mean age ± SD, 47.77 ± 11.62 years) were used in the subsequent data analyses.

**Table S1. Demographic information of the participants who performed the reward-seeking ($n$ = 939) and loss-avoidance ($n$ = 961) tasks**

| | Reward-seeking task | Loss-avoidance task |
|---|---|---|
| Age (mean ± SD years) | 47.90 ± 11.70 | 47.64 ± 11.54 |
| Sex (% of the females) | 52.61% | 52.65% |
| SES (mean ± SD) | 21.79 ± 7.08 | 21.68 ± 6.87 |
| Education (mean ± SD) | 4.53 ± 1.82 | 4.51 ± 1.81 |

SES, socioeconomic status measured by the questionnaire [3]. Education was coded as junior high school diploma: 1; high school diploma: 2; technical school diploma: 3; vocational school diploma: 4; associate degree (community college diploma): 5; bachelor's degree: 6; and a master's or doctorate degree: 7.

**Decision-making tasks: timeline of each trial.**

At the beginning of each trial, participants were required to choose among three options by clicking one of the fractal stimuli within 6 s (Decision phase; Fig. 1a). In this task, in every trial the three options (*green*, *red,* and *blue*) were positioned in the left, middle, and right of the screen, respectively. After making a response, the chosen option was highlighted by a yellow frame (Confirmation phase, 1 s). The outcome of the choice was then revealed to the participants (Feedback phase, 1.5 s). If no response was made in the decision phase, the remaining phases were skipped, and the participants moved to the next trial.

**Reward payment**

**Reward-seeking task.** In addition to a participation fee of 500 Japanese *yen*, participants obtained an extra reward depending on their task performance. The amount of performance-based rewards was determined as follows: at the end of the experiment, the computer randomly selected one trial, and the outcome of the trial was implemented. If the participant received a reward in that trial, they obtained 100 *yen*. Because the participants did not know which trial would be selected for the monetary reward, they should have treated each trial as if it was being implemented. The reward they earned was paid with a coupon that could be

used in popular Japanese online stores, including Amazon Japan (https://www.amazon.co.jp/) and Rakuten Ichiba (https://www.rakuten.co.jp/).

**Loss-avoidance task.** Participants were endowed with a participation fee of 600 Japanese *yen*. Depending on their task performance, they lost an amount of up to 100 *yen* (the loss was subtracted from the initial endowment). As in the reward-seeking task, the computer randomly selected one trial at the end of the experiment, and the outcome of that trial was implemented.

### Questionnaires

After the decision-making task, the participants were administered the Japanese versions of the following questionnaires: Schizotypal Personality Questionnaire Brief [4,5], Obsessive-Compulsive Inventory [6], Self-Rating Depression Scale [7,8], State-Trait Anxiety Inventory [9,10], and Barratt Impulsivity Scale[11,12]. Questionnaires were presented in the aforementioned order to all the participants to ensure that any individual differences in self-reported psychiatric symptoms were not attributed to order-effects. To identify participants who did not respond to the questionnaires seriously, a catch question was included in the Obsessive–Compulsive Inventory: ‘If you have carefully read the questions so far, please select 'a little' as your answer’.

**Table S2. Total scores of the questionnaires (*n* = 1900)**

| | Mean ± SD | Min | Max |
|---|---|---|---|
| SPQB | 6.38 ± 4.36 | 0 | 22 |
| OCI | 21.19 ± 22.80 | 0 | 149 |
| SDS | 41.00 ± 8.62 | 20 | 73 |
| STAI | 85.39 ± 20.45 | 40 | 158 |
| BIS | 57.37 ± 14.39 | 10 | 117 |

SPQB, Schizotypal Personality Questionnaire Brief; OCI, Obsessive-Compulsive Inventory; SDS, Self-Rating Depression Scale; STAI, State-Trait Anxiety Inventory; and BIS, Barratt Impulsivity Scale.

**Table S3. Loadings of each item for the three factors (*n* = 1900)**

| Questionnaire | Item | F1 loading | F2 loading | F3 loading |
|---|---|---|---|---|
| SPQB | 1 | 0.31 | -0.08 | 0.19 |
| | 2 | 0.37 | -0.16 | 0.09 |
| | 3 | 0.37 | 0.01 | 0.17 |
| | 4 | 0.37 | 0.03 | 0.14 |
| | 5 | 0.39 | -0.30 | 0.11 |
| | 6 | 0.22 | -0.02 | 0.25 |
| | 7 | 0.41 | 0.06 | 0.03 |
| | 8 | 0.31 | 0.12 | 0.21 |
| | 9 | 0.38 | 0.21 | 0.07 |
| | 10 | 0.44 | 0.07 | 0.07 |
| | 11 | 0.17 | 0.28 | 0.06 |
| | 12 | 0.35 | -0.23 | 0.07 |
| | 13 | 0.35 | -0.14 | 0.20 |
| | 14 | 0.22 | 0.18 | -0.03 |
| | 15 | 0.07 | 0.34 | -0.06 |
| | 16 | 0.45 | -0.03 | 0.02 |
| | 17 | 0.27 | 0.22 | 0.09 |
| | 18 | 0.26 | 0.30 | 0.02 |
| | 19 | 0.33 | 0.06 | 0.14 |
| | 20 | 0.18 | 0.25 | 0.18 |
| | 21 | 0.09 | 0.33 | 0.08 |
| | 22 | 0.18 | 0.15 | -0.13 |
| OCI | 1 | 0.56 | 0.15 | 0.03 |
| | 2 | 0.58 | 0.04 | 0.01 |
| | 3 | 0.61 | -0.03 | 0.07 |
| | 4 | 0.65 | 0.03 | -0.09 |
| | 5 | 0.67 | 0.05 | 0.00 |
| | 6 | 0.53 | 0.07 | 0.02 |
| | 7 | 0.74 | 0.06 | -0.14 |
| | 8 | 0.63 | -0.02 | -0.10 |
| | 9 | 0.70 | -0.02 | -0.13 |
| | 10 | 0.67 | -0.05 | -0.19 |

| | | | | |
|---|---|---|---|---|
| | 11 | 0.49 | -0.07 | 0.13 |
| | 12 | 0.63 | -0.01 | 0.09 |
| | 13 | 0.55 | 0.05 | 0.10 |
| | 14 | 0.67 | -0.03 | -0.06 |
| | 15 | 0.73 | 0.00 | -0.14 |
| | 16 | 0.72 | -0.12 | -0.02 |
| | 17 | 0.67 | 0.01 | 0.11 |
| | 18 | 0.68 | -0.06 | 0.01 |
| | 19 | 0.77 | -0.01 | -0.13 |
| | 20 | 0.60 | -0.06 | 0.06 |
| | 21 | 0.74 | -0.07 | -0.15 |
| | 22 | 0.70 | -0.04 | -0.09 |
| | 23 | 0.77 | -0.09 | -0.13 |
| | 24 | 0.71 | 0.01 | -0.02 |
| | 25 | 0.73 | -0.13 | 0.00 |
| | 26 | 0.75 | -0.02 | -0.05 |
| | 27 | 0.66 | -0.03 | -0.06 |
| | 28 | 0.59 | 0.12 | 0.13 |
| | 29 | 0.78 | -0.04 | -0.10 |
| | 30 | 0.71 | 0.10 | -0.04 |
| | 31 | 0.76 | -0.08 | -0.04 |
| | 32 | 0.73 | -0.08 | -0.02 |
| | 33 | 0.67 | 0.10 | 0.04 |
| | 34 | 0.58 | -0.02 | -0.07 |
| | 35 | 0.70 | -0.01 | -0.02 |
| | 36 | 0.75 | -0.03 | -0.02 |
| | 37 | 0.76 | 0.01 | -0.01 |
| | 38 | 0.75 | -0.04 | -0.09 |
| | 39 | 0.60 | -0.08 | -0.04 |
| | 40 | 0.73 | -0.07 | -0.11 |
| | 41 | 0.76 | 0.02 | -0.08 |
| | 42 | 0.72 | -0.06 | -0.11 |
| SDS | 1 | 0.37 | 0.48 | -0.03 |
| | 2 | -0.17 | 0.40 | -0.02 |

| | | | | |
|---|---|---|---|---|
| | 3 | 0.33 | 0.26 | 0.07 |
| | 4 | 0.29 | 0.26 | -0.07 |
| | 5 | 0.11 | 0.10 | 0.12 |
| | 6 | -0.13 | 0.15 | -0.02 |
| | 7 | 0.33 | -0.14 | 0.11 |
| | 8 | 0.15 | 0.14 | 0.07 |
| | 9 | 0.40 | 0.16 | 0.05 |
| | 10 | 0.23 | 0.35 | 0.04 |
| | 11 | 0.05 | 0.67 | -0.02 |
| | 12 | 0.12 | 0.45 | 0.08 |
| | 13 | 0.44 | 0.08 | 0.16 |
| | 14 | -0.07 | 0.70 | -0.03 |
| | 15 | 0.30 | 0.37 | 0.03 |
| | 16 | 0.00 | 0.56 | -0.02 |
| | 17 | -0.10 | 0.60 | 0.17 |
| | 18 | -0.06 | 0.76 | -0.03 |
| | 19 | 0.25 | 0.36 | 0.13 |
| | 20 | -0.03 | 0.74 | 0.00 |
| STAI | 1 | 0.11 | 0.62 | 0.00 |
| | 2 | 0.12 | 0.71 | -0.04 |
| | 3 | 0.41 | 0.18 | 0.04 |
| | 4 | 0.32 | 0.31 | 0.02 |
| | 5 | -0.10 | 0.70 | -0.07 |
| | 6 | 0.43 | 0.19 | 0.08 |
| | 7 | 0.39 | 0.39 | -0.03 |
| | 8 | 0.01 | 0.76 | -0.05 |
| | 9 | 0.31 | 0.50 | -0.03 |
| | 10 | -0.01 | 0.79 | -0.08 |
| | 11 | -0.10 | 0.75 | 0.02 |
| | 12 | 0.38 | 0.26 | 0.04 |
| | 13 | 0.33 | 0.36 | -0.01 |
| | 14 | 0.46 | 0.26 | -0.01 |
| | 15 | 0.04 | 0.74 | -0.12 |
| | 16 | -0.04 | 0.84 | -0.07 |

| | | | | |
|---|---|---|---|---|
| | 17 | 0.34 | 0.40 | -0.03 |
| | 18 | 0.49 | 0.15 | 0.02 |
| | 19 | -0.22 | 0.74 | -0.17 |
| | 20 | -0.12 | 0.76 | -0.07 |
| | 21 | -0.02 | 0.73 | -0.13 |
| | 22 | 0.18 | 0.34 | 0.08 |
| | 23 | 0.30 | 0.31 | 0.10 |
| | 24 | 0.25 | 0.23 | 0.02 |
| | 25 | 0.19 | 0.34 | 0.08 |
| | 26 | 0.00 | 0.76 | -0.06 |
| | 27 | -0.04 | 0.76 | 0.05 |
| | 28 | 0.22 | 0.33 | 0.15 |
| | 29 | 0.46 | 0.27 | 0.05 |
| | 30 | 0.02 | 0.75 | -0.12 |
| | 31 | 0.45 | 0.21 | -0.08 |
| | 32 | 0.18 | 0.50 | 0.11 |
| | 33 | -0.01 | 0.81 | -0.06 |
| | 34 | 0.08 | 0.18 | 0.26 |
| | 35 | 0.26 | 0.53 | 0.03 |
| | 36 | -0.02 | 0.81 | -0.06 |
| | 37 | 0.41 | 0.25 | 0.10 |
| | 38 | 0.32 | 0.34 | 0.09 |
| | 39 | -0.09 | 0.64 | 0.04 |
| | 40 | 0.44 | 0.23 | 0.12 |
| BIS | 1 | -0.39 | 0.28 | 0.45 |
| | 2 | -0.10 | -0.13 | 0.67 |
| | 3 | -0.09 | -0.45 | 0.40 |
| | 4 | -0.13 | -0.66 | 0.37 |
| | 5 | -0.15 | -0.05 | 0.55 |
| | 6 | 0.38 | 0.05 | 0.06 |
| | 7 | -0.41 | 0.21 | 0.39 |
| | 8 | -0.09 | 0.29 | 0.34 |
| | 9 | -0.07 | 0.42 | 0.30 |
| | 10 | -0.12 | 0.29 | 0.20 |

| | | | |
|---|---|---|---|
| 11 | 0.18 | 0.23 | 0.20 |
| 12 | -0.35 | 0.12 | 0.50 |
| 13 | -0.27 | 0.14 | 0.15 |
| 14 | 0.01 | -0.06 | 0.59 |
| 15 | -0.25 | 0.37 | 0.12 |
| 16 | 0.07 | 0.02 | 0.34 |
| 17 | 0.05 | -0.12 | 0.74 |
| 18 | -0.02 | 0.04 | 0.67 |
| 19 | 0.13 | -0.07 | 0.67 |
| 20 | -0.04 | 0.55 | 0.21 |
| 21 | 0.19 | -0.13 | 0.32 |
| 22 | 0.04 | -0.11 | 0.63 |
| 23 | 0.14 | 0.05 | 0.40 |
| 24 | 0.14 | -0.12 | 0.49 |
| 25 | 0.11 | -0.08 | 0.55 |
| 26 | 0.19 | -0.01 | 0.52 |
| 27 | 0.02 | -0.31 | 0.30 |
| 28 | 0.20 | 0.01 | 0.44 |
| 29 | 0.11 | -0.17 | 0.04 |
| 30 | -0.12 | 0.57 | 0.00 |

F1, compulsive behaviour and intrusive thought (CIT); F2, anxiety-depression (AD); and F3, impulsivity (IM). SPQB, Schizotypal Personality Questionnaire Brief; OCI, Obsessive-Compulsive Inventory; SDS, Self-Rating Depression Scale; STAI, State-Trait Anxiety Inventory; and BIS, Barratt Impulsivity Scale.

## Method S2: Data Analysis

The data were analysed using MATLAB R2020a and R (version 3.6.3) on a MacBook Pro (Retina 5K, 13-inch, 2018; Mac OS X 10.14.6).

### Psychiatric dimensions and decision-making processes: mixed-effect regression analysis

To examine the effects of past rewards (no-losses) and choices on participants' current behaviour and the modulation of these effects by psychiatric factors, we conducted a Generalised Linear Mixed Model (GLMM) analysis using the MATLAB R2020a function, *fitglme*, with the restricted maximum pseudo-likelihood estimation. Based on previous studies using the three-armed bandit task [13,14], we performed three separate logistic regression models, one for each choice option (*X*, *Y,* and *Z*).

In the reward-seeking task, for one option *X*, the GLMM was defined in the Wilkinson notation as follows: logit P(choice = *X*) ~ 1 + ($R_{t\text{-}1}$ + $R_{t\text{-}2}$ + $R_{t\text{-}3}$ + $R_{t\text{-}4}$ + $C_{t\text{-}1}$ + $C_{t\text{-}2}$ + $C_{t\text{-}3}$ + $C_{t\text{-}4}$) * (*CIT* + *AD* + *IM* + *age* + *sex* + *education* + *ses*) + (1 + $R_{t\text{-}1}$ + $R_{t\text{-}2}$ + $R_{t\text{-}3}$ + $R_{t\text{-}4}$ + $C_{t\text{-}1}$ + $C_{t\text{-}2}$ + $C_{t\text{-}3}$ + $C_{t\text{-}4}$ | participant),

where $R_{t-\tau}$ and $C_{t-\tau}$ denote recent past rewards and recent past choices, respectively (trials *t*-1, *t*-2, *t*-3, and *t*-4), and *CIT, AD* and *IM* represent the psychiatric factors. As per previous studies [13,14], $R_{t-\tau}$ was coded as 1 if the participant chose *X* and obtained a reward on trial $t-\tau$, -1 if they chose *Y* or *Z* and obtained a reward, and 0 if there was no reward. $C_{t-\tau}$ was coded as 1 if the participant chose *X* on trial $t-\tau$, and -1 otherwise. The term (. | participants) indicates within-participant variables considered as random-effects (i.e., allowed to vary between participants). The model provided a set of regression coefficients and covariances. Here, we were interested in the fixed effects of past rewards and past choices, and their interactions with psychiatric factors. The total effect of past rewards over the past *four* trials was derived by $\beta(R)=\sum_{i=1}^{4}\beta(R_{t-i})$, where $\beta(R_{t-i})$ denotes the regression coefficient of the variable $R_{t-i}$; and the variance of the total effect was computed by $\sigma^2(R)=\sum_{i=1}^{4}\sum_{j=1}^{4}cov(R_{t-i},R_{t-j})$, where $cov(R_{t-i},R_{t-j})$ denotes the covariance of the regression coefficients, $\beta(R_{t-i})$ and $\beta(R_{t-j})$. The total effect of past choices and its

variance, as well as the total interaction effects and their variances, were derived similarly. In the loss-avoidance task, we performed the same GLMM, treating 'no-loss' and 'loss' as 'reward' and 'no-reward', respectively.

The GLMMs for the two other options, *Y* and *Z*, were defined in the same manner, providing the total effects of past rewards, past choices, and their interactions with psychiatric factors. The mean effect of past rewards over the three models was derived by the variance-weighted mean [15]: $\frac{\beta_X(R)/\sigma_X^2(R)+\beta_Y(R)/\sigma_Y^2(R)+\beta_Z(R)/\sigma_Z^2(R)}{1/\sigma_X^2(R)+1/\sigma_Y^2(R)+1/\sigma_Z^2(R)}$, where subscripts denote each of the three models. The variance of the mean was given by $\frac{1}{1/\sigma_X^2(R)+1/\sigma_Y^2(R)+1/\sigma_Z^2(R)}$ (see [15]). Based on the mean effect and its variance (standard deviation), we tested the statistical significance of the effect using a two-tailed *t*-test with FDR multiple-comparison correction [16] (see Fig. 4 legends). The same procedure was applied to the statistical tests that analysed the effect of past choices and the effects of the interactions with psychiatric factors.

**Psychiatric dimensions and decision-making processes: model-based analysis**

We constructed computational models and fitted them to the participants' choice behaviours in the decision-making tasks.

**RL1a.** The first model was a conventional Reinforcement Learning (RL) model, termed Q-learning [17]. In this model, on each trial, an agent makes a choice depending on the value of each option. The choice probability of an option *X* is given by the following equation:

$$q(X) = {exp\big(\beta Q(X)\big)}\Big/{\big[exp\big(\beta Q(X)\big) + exp\big(\beta Q(Y)\big) + exp\big(\beta Q(Z)\big)\big]},$$

where *Q* denotes option values and the parameter $\beta \in [0, \infty)$ governs the degree of stochasticity in the choices (termed *inverse temperature*) [17]. Once a choice is made and the reward outcome is revealed, the agent updates the value of the chosen option (i.e., learning). Suppose an option *X* is chosen, then the value of the option *X* is updated as follows:

$$Q(X) \leftarrow Q(X) + \alpha\big(R - Q(X)\big),$$

where $R$ denotes reward outcome (coded 1 for reward and 0 for no reward in the reward-seeking task, and coded 1 for no-loss and 0 for loss in the loss-avoidance task) and the parameter $\alpha \in [0,1]$ is the *learning rate* [17]. In the initial experimental trial, option values were set at 0.5 (as the agent had no knowledge of the reward/no-loss probabilities).

The other models below are variants of the conventional RL model (i.e., RL1a).

**RL1b.** This model is almost identical to RL1a but includes 'forgetting'. That is, the values of the unchosen options are forgotten (i.e., decay with time) [18–21]. In other words, on each trial, an agent updates not only the value of the chosen option but also the values of the unchosen options. Specifically, values of the unchosen options, $Y$ and $Z$, are updated as follows:

$Q(Y) \leftarrow Q(Y) - \alpha_F Q(Y)$, and $Q(Z) \leftarrow Q(Z) - \alpha_F Q(Z)$,

where $\alpha_F \in [0,1]$ denotes the *forgetting rate*. Note that the value of the chosen option is also updated as in RL1a.

**RL2a.** In this model, an agent considers their own choice-trace in the decision-making. The choice-trace of each option, which quantifies how often the option was recently chosen, is updated according to the following rule:

$$C(X) \leftarrow C(X) + \alpha_C\big(I(X) - C(X)\big),$$

where $I(\cdot)$ is 1 if the option is chosen and 0 otherwise, and $\alpha_C \in [0,1]$ is the *choice-trace decay rate*. Note that the choice-traces of the other two options are updated by the same rule and that the initial choice-traces are set at *zero*. The choice-traces function in decision-making as follows:

$$q(X) = \frac{exp\big(\beta Q(X)+\gamma C(X)\big)}{\big[exp\big(\beta Q(X)+\gamma C(X)\big)+exp\big(\beta Q(Y)+\gamma C(Y)\big)+exp\big(\beta Q(Z)+\gamma C(Z)\big)\big]},$$

where $\gamma \in (-\infty, \infty)$ denotes the *weight of the choice-traces*. The agent considers both reward values and choice-traces in the decision-making. Individuals with positive choice-trace weights are likely to repeat a recently selected choice. Conversely, individuals with negative choice-trace weights tend to avoid a recently chosen option.

**RL2b.** This model is almost identical to RL2a but includes value-forgetting. That is, values of the unchosen options are forgotten.

**RL3a.** This model has differential learning rates for reward outcomes. Specifically, the value of the chosen option is updated as follows:

$$Q(X) \leftarrow \begin{cases} Q(X) + \alpha_+\big(R - Q(X)\big) & if\ R = 1 \\ Q(X) + \alpha_-\big(R - Q(X)\big) & if\ R = 0 \end{cases},$$

where $\alpha_+ \in [0,1]$ and $\alpha_- \in [0,1]$ depict the learning rates for rewarding and non-rewarding outcomes, respectively. The process of decision-making is identical to that of RL1a.

**RL3b.** This model is almost identical to RL3a but includes value-forgetting.

**RL4a.** This model has an adaptive learning rate [22]. That is, the value of the chosen option is updated at trial $t$ as follows: $Q_{t+1}(X) \leftarrow Q_t(X) + \kappa\alpha_t\delta_t$, where $\delta_t = R_t - Q_t(X)$ denotes the reward prediction error. Critically, the learning rate, $\kappa\alpha_t$, depends on the (absolute) reward prediction error at the previous trial $t - 1$: $\alpha_t \leftarrow \eta|\delta_{t-1}| + (1 - \eta)\alpha_{t-1}$, where $\eta \in [0,1]$ denotes the weight of the previous reward prediction error and $\kappa \in [0,1]$ is the constant term. At the initial trial, the learning rate is $\kappa\alpha_1$ where $\alpha_1 \in [0,1]$.

**RL4b.** This model is almost identical to RL4a but includes value-forgetting.

**Procedures of model fitting and comparison.** To fit the computational models to each participant's choice data, we employed a maximum *a posteriori* (MAP) approach incorporating prior beliefs about the parameter values (rather than a maximum likelihood approach in which variances of the parameter estimates are known to be inflated)[23]. Based on a previous study[24], we set prior distributions of the value learning rates, $\alpha$, $\alpha_+$, $\alpha_-$, and $\alpha_1$ at *Beta* (2,2) and the inverse temperature, $\beta$, at *Gamma* (2,3). For the forgetting rate, $\alpha_F$, choice-trace decay rate, $\alpha_C$, and the other parameters ($\kappa$, $\eta$), we used flatter prior distributions, *Beta* (1.2,1.2), as we did not have strong hypotheses about the parameter values *a priori*. A prior distribution of the choice-trace weight was set at *Norm* (0,4). For each model and participant, we obtained a MAP estimate using the MATLAB function, *fmincon*, and then computed log model evidence using Laplace approximations [23]. Here, note that log model evidence implicitly penalises models with more free parameters as compared to those with fewer parameters. Each model's log model evidence was finally fed into Bayesian model selection[25] to compare the goodness of fit of the competing models. As a robustness check, we assessed different prior distributions for learning rates (*Beta* (3, 2) and *Beta*

(3,1.5)), revealing that the winning model did not change (i.e., Exceedance probability of RL2b is 1.00 for all the cases).

**Confusion matrix of model fitting.** Before fitting these models to the actual data, we verified the identifiability of the models given the current experimental settings [26]. We tested whether each of the models captured unique behavioural patterns. To this end, we constructed a 'confusion matrix' [26] based on simulated data. If the models were perfectly identifiable, simulation data generated by one model should be best explained by the same model rather than other models, and the confusion matrix should thus be the identity matrix. In this analysis, we first generated simulation data of 1,000 agents for each model. The number of agents was almost the same to the number of actual participants (see Participants). Decision parameters of each agent were sampled from the prior distributions (see Procedures of model fitting and comparison). We then formed a confusion matrix based on exceedance probabilities of Bayesian model selection[25]. As a result, we identified that exceedance probabilities in the diagonal cells were almost *one* (Fig. S4a), suggesting that these models were identifiable given the current experimental settings[26].

**Parameter recovery analysis and replication of the original results based on simulation data.** After obtaining the model fitting results, for further validation, we generated simulation data of 939 and 961 agents, whose decision parameters were extracted by the best-fit model and parameters [26]. For the simulated data, we aimed to confirm that the parameter values could be recovered by model fitting and that the original results can be replicated. In the analyses, the parameter values were successfully recovered (Fig. S4jk) and the results of regression-based analyses on simulation data were highly consistent with those on the actual data (Fig. S4b-i), implying that the model and parameters reliably captured meaningful computational processes and their individual differences [26].

## SUPPLEMENTAL MATERIAL: References


1. Gillan CM, Kosinski M, Whelan R, Phelps EA, Daw ND. Characterizing a psychiatric symptom dimension related to deficits in goal-directed control. *Elife*. 2016; 5: e11305.

2. Maniaci MR, Rogge RD. Caring about carelessness: Participant inattention and its effects on research. *J Res Pers*. 2014; 48: 61–83.

3. Yanagisawa K, Masui K, Furutani K, Nomura M, Yoshida H, Ura M. Family socioeconomic status modulates the coping-related neural response of offspring. *Soc Cogn Affect Neur*. 2013; 8: 617–22.

4. Raine A, Benishay D. The SPQ-B: A Brief Screening Instrument for Schizotypal Personality Disorder. *J Pers Disord*. 1995; 9: 346–55.

5. Ito S, Obu S, Ota M, Takao T, Sakamoto S. Reliability and validity of the Japanese version of SPQ-B (Schizotypal Personality Questionnaire Brief). *Jpn. Bull. Soc. Psychiatry*. 2008; : 168–76.

6. Ishikawa R, Kobori O, Shimizu E. Development and validation of the Japanese version of the obsessive-compulsive inventory. *Bmc Res Notes*. 2014; 7: 306.

7. ZUNG WWK. A Self-Rating Depression Scale. *Arch Gen Psychiat*. 1965; 12: 63–70.

8. Fukuda K, Kobayashi S. A Study of Self-Rating Depression Scale. *Psychiatria et neurologia Japonica (in Japanese)*. 1973; 75: 673–9.

9. D. S C, L. G R, E. L R. *Manual for the State-Trait Anxiety Inventory (Self-Evaluation Questionnaire)*. Consulting Psychologists Press; 1970.

10. Shimizu H, Imaei K. Japanese version of the STATE-TRAIT ANXIETY INVENTORY (for college students). *The Japanese Journal of Educational Psychology (in Japanese)*. 1981; 29: 348–53.

11. Patton JH, Stanford MS, Barratt ES. Factor structure of the barratt impulsiveness scale. *J Clin Psychol*. 1995; 51: 768–74.

12. Kobashi M, Ida M. Making the Revised Version of Barratt Impulsiveness Scale 11th in Japanese: A Study on Reliability and Validity. *The journal of psychology Rissho University (in Japanese)*. 2013; 4: 53–61.

13. Walton ME, Behrens TEJ, Buckley MJ, Rudebeck PH, Rushworth MFS. Separable Learning Systems in the Macaque Brain and the Role of Orbitofrontal Cortex in Contingent Learning. *Neuron*. 2010; 65: 927–39.

14. Noonan MP, Chau BKH, Rushworth MFS, Fellows LK. Contrasting Effects of Medial and Lateral Orbitofrontal Cortex Lesions on Credit Assignment and Decision-Making in Humans. *J Neurosci*. 2017; 37: 7023–35.

15. Leo WR. *Techniques for Nuclear and Particle Physics Experiments*. Springer-Verlag; 1994.

16. Benjamini Y, Hochberg Y. Controlling the False Discovery Rate: A Practical and Powerful Approach to Multiple Testing. *J Royal Statistical Soc Ser B Methodol*. 1995; 57: 289–300.

17. Sutton RS, Barto AG. *Reinforcement Learning: An Introduction*. MIT Press; 1998.

18. Ito M, Doya K. Validation of Decision-Making Models and Analysis of Decision Variables in the Rat Basal Ganglia. *J Neurosci*. 2009; 29: 9861–74.

19. Katahira K, Yuki S, Okanoya K. Model-based estimation of subjective values using choice tasks with probabilistic feedback. *J Math Psychol*. 2017; 79: 29–43.

20. Toyama A, Katahira K, Ohira H. A simple computational algorithm of model-based choice preference. *Cognitive Affect Behav Neurosci*. 2017; 17: 764–83.

21. Kato A, Morita K. Forgetting in Reinforcement Learning Links Sustained Dopamine Signals to Motivation. *Plos Comput Biol*. 2016; 12: e1005145.

22. Li J, Schiller D, Schoenbaum G, Phelps EA, Daw ND. Differential roles of human striatum and amygdala in associative learning. *Nat Neurosci*. 2011; 14: 1250–2.

23. Daw ND. Trial-by-trial data analysis using computational models. **In**: Delgado MR, Phelps Elizabeth A., Robbins Trevor W. (eds.). undefined. Oxford Scholarship Online; 2011.

24. Niv Y, Edlund JA, Dayan P, O'Doherty JP. Neural Prediction Errors Reveal a Risk-Sensitive Reinforcement-Learning Process in the Human Brain. *J Neurosci*. 2012; 32: 551–62.

25. Stephan KE, Penny WD, Daunizeau J, Moran RJ, Friston KJ. Bayesian model selection for group studies. *Neuroimage*. 2009; 46: 1004–17.

26. Wilson RC, Collins A. Ten simple rules for the computational modeling of behavioral data. *undefined*.

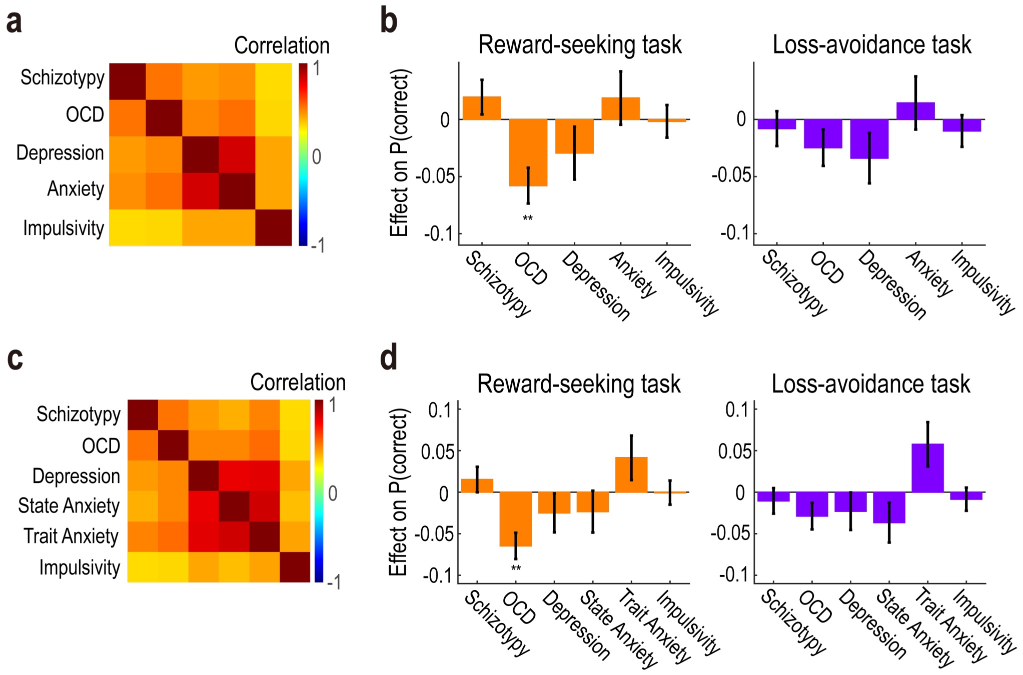


**Fig. S1: Supplementary analysis on the total scores of the questionnaires**

(a) Cross-correlation of the five questionnaires' total scores.

(b) Effects of the questionnaires' total scores on the proportion of correct choices. A correct choice was defined as the selection of the option with the highest reward (or no-loss) probability in a given trial. The mean and SEM of the effects were estimated with a generalised linear mixed-effect model. *Left*, reward-seeking task (mean ± SEM, $N$ = 465,435); and *right*, loss-avoidance task (mean ± SEM, $N$ = 476,115). **$P$ < 0.01, FDR-corrected by the number of tests (i.e., 5) in two-tailed $t$-test (for the *reward-seeking* task, effect of Schizotypy: corrected $P$ = 0.445 and uncorrected $P$ = 0.235; OCD: corrected and uncorrected $Ps$ < 0.001; Depression: corrected $P$ = 0.445 and uncorrected $P$ = 0.267; Anxiety: corrected $P$ = 0.669 and uncorrected $P$ = 0.535; Impulsivity: corrected and uncorrected $Ps$ = 0.952; for the *loss-avoidance* task, Schizotypy: corrected and uncorrected $Ps$ = 0.660; OCD: corrected $P$ = 0.453 and uncorrected $P$ = 0.091; Depression: corrected $P$ = 0.660 and uncorrected $P$ = 0.346; Anxiety: corrected $P$ = 0.660 and uncorrected $P$ = 0.539; Impulsivity: corrected $P$ = 0.660 and uncorrected $P$ = 0.635; for the loss-avoidance task:).

(c) Cross-correlation of the six questionnaires' total scores. Total scores of the state and trait anxiety were derived separately.

(d) Effects of the questionnaires' total scores on the proportion of correct choices. Same

format as in Fig. S1b except that the total scores of the state and trait anxiety were obtained separately. **$P < 0.01$, FDR-corrected by the number of tests (i.e., 6) in two-tailed *t*-test (for the *reward-seeking* task, effect of Schizotypy: corrected $P$ = 0.422 and uncorrected $P$ = 0.320; OCD: corrected and uncorrected *Ps* < 0.001; Depression: corrected $P$ = 0.422 and uncorrected $P$ = 0.287; State Anxiety: corrected $P$ = 0.422 and uncorrected $P$ = 0.352; Trait Anxiety: corrected $P$ = 0.366 and uncorrected $P$ = 0.122; Impulsivity: corrected and uncorrected *Ps* = 0.973; for the *loss-avoidance* task, Schizotypy: corrected $P$ = 0.546 and uncorrected $P$ = 0.500; OCD: corrected $P$ = 0.217 and uncorrected $P$ = 0.072; Depression: corrected $P$ = 0.466 and uncorrected $P$ = 0.311; State Anxiety: corrected $P$ = 0.253 and uncorrected $P$ = 0.127; Trait Anxiety: corrected $P$ = 0.183 and uncorrected $P$ = 0.031; Impulsivity: corrected and uncorrected *Ps* = 0.546).

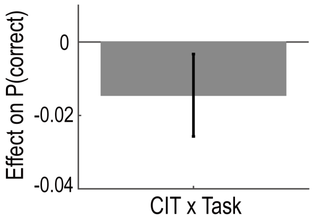


**Fig. S2: Supplementary analysis of psychiatric factors (dimensions) and decision-making performance**

Interaction effect on the proportion of correct choices (mean ± SEM, *N* = 941,550). The mean and SEM of the interaction effect between CIT and Task (reward-seeking vs. loss-avoidance) was estimated with a generalised linear mixed-effect model (GLMM) (effect of CIT x Task: *P* = 0.196). CIT, ‘compulsive behaviour and intrusive thought’.

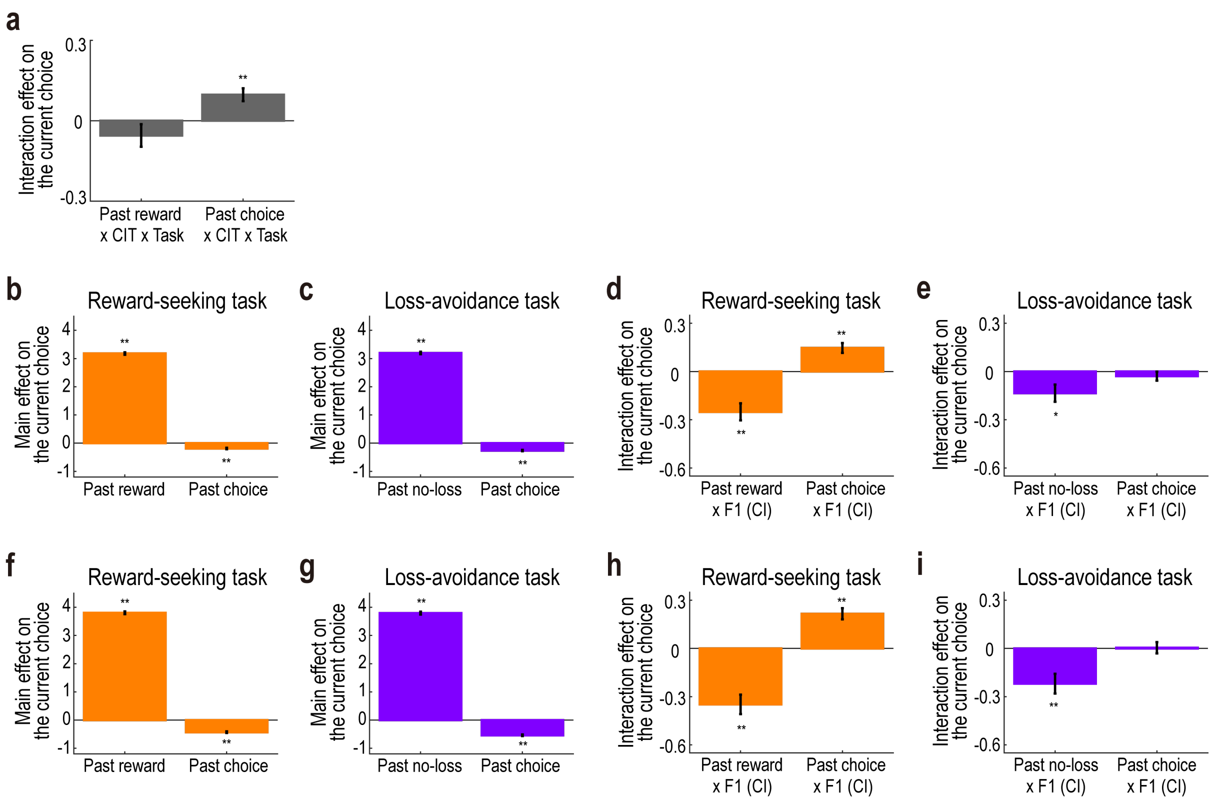


**Fig. S3: Supplementary analysis of psychiatric factors (dimensions) and decision-making processes**

(a) Three-way interaction effects on the current choice (mean ± SEM, $N$ = 941,550). *Left*, interaction effect among past reward (no-loss), CIT and task (reward-seeking vs. loss-avoidance); and *right*, interaction effect among past choice, CIT and task. **$P$ < 0.01, FDR-corrected by the number of tests (i.e., 2) in two-tailed $t$-tests (Past reward x CIT x Task: corrected and uncorrected $P$s = 0.194; Past choice x CIT x Task: corrected and uncorrected $P$s < 0.001). CIT, ‘compulsive behaviour and intrusive thought’.

(b)-(e) GLMM analysis considering the reward and choice history of *two* previous trials. Same format as in Fig. 4 (for the *reward-seeking* task, Past reward: corrected and uncorrected $P$s < 0.001; Past choice: corrected and uncorrected $P$s < 0.001; Past reward x CIT: corrected and uncorrected $P$s < 0.001; Past choice x CIT: corrected and uncorrected $P$s < 0.001; and for the *loss-avoidance* task, Past no-loss: corrected and uncorrected $P$s < 0.001; Past choice: corrected and uncorrected $P$s < 0.001; Past no-loss x CIT: corrected $P$ = 0.024 and uncorrected $P$ = 0.012; Past choice x CIT: corrected and uncorrected $P$s = 0.303).

(f)-(i) GLMM analysis that considering the reward and choice history of *six* previous trials. Same format as in Fig. 4 (for the *reward-seeking* task, Past reward: corrected and

uncorrected *Ps* < 0.001; Past choice: corrected and uncorrected *Ps* < 0.001; Past reward x CIT: corrected and uncorrected *Ps* < 0.001; Past choice x CIT: corrected and uncorrected *Ps* < 0.001; and for the *loss-avoidance* task, Past no-loss: corrected and uncorrected *Ps* < 0.001; Past choice: corrected and uncorrected *Ps* < 0.001; Past no-loss x CIT: corrected and uncorrected *Ps* < 0.001; Past choice x CIT: corrected and uncorrected *Ps* = 0.909).

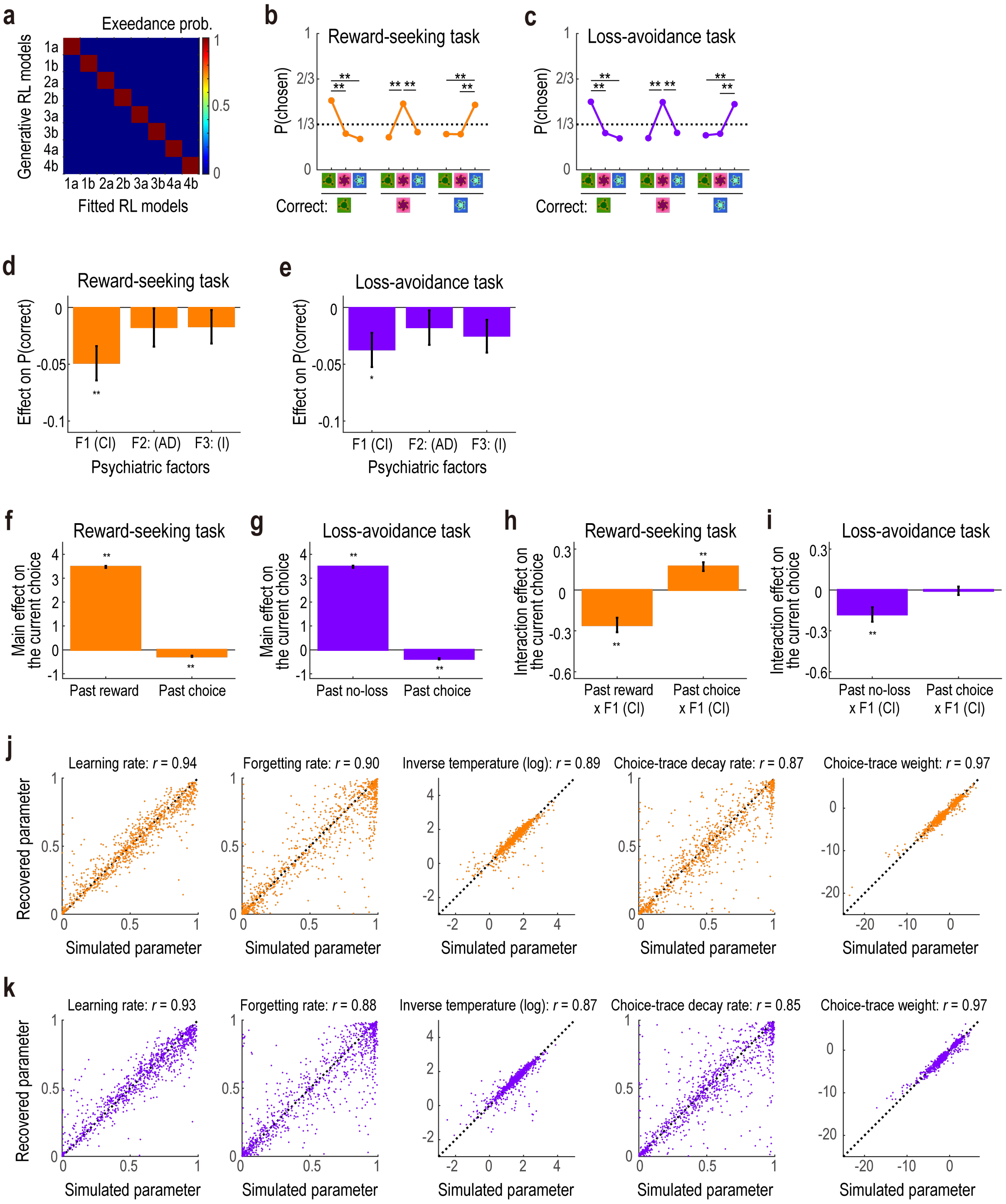


**Fig. S4: Validation of computational models and model-fitting procedure based on simulation data**

(a) Confusion matrix to evaluate the performance of our Bayesian model selection. Each row denotes exceedance probabilities of the eight competing models on the data generated by the corresponding model. For example, the first row depicts exceedance probabilities of the eight models on the data generated by the first model (i.e., RL1a). The diagonal elements

are close to 1, indicating that each of these models is identifiable by the model selection procedure.

(bc) Replication of results in Fig. 1ef. We simulated the data based on the best-fit model (i.e., RL2b) and parameters, and then replicated the original results on the simulated data. Same format as in Fig. 1ef (corrected and uncorrected *Ps* < 0.001 for all the comparisons).

(de) Replication of the results in Fig. 3. Same format as in Fig. 3 (for the *reward-seeking* task, effect of CIT: corrected *P* = 0.004 and uncorrected *P* = 0.001; AD: corrected and uncorrected *Ps* = 0.293; and IM: corrected *P* = 0.293 and uncorrected *P* = 0.248; and for the *loss-avoidance* task, CIT: corrected *P* = 0.038 and uncorrected *P* = 0.013; AD: corrected and uncorrected *Ps* = 0.241; and IM: corrected *P* = 0.119 and uncorrected *P* = 0.079).

(f-i) Replication of the results in Fig. 4. Same format as in Fig. 4 (for the *reward-seeking* task, Past reward: corrected and uncorrected *Ps* < 0.001; Past choice: corrected and uncorrected *Ps* < 0.001; Past reward x CIT: corrected and uncorrected *Ps* < 0.001; Past choice x CIT: corrected and uncorrected *Ps* < 0.001; and for the *loss-avoidance* task, Past no-loss: corrected and uncorrected *Ps* < 0.001; Past choice: corrected and uncorrected *Ps* < 0.001; Past no-loss x CIT: corrected and uncorrected *Ps* < 0.001; Past choice x CIT: corrected and uncorrected *Ps* = 0.843).

(j) Parameter recovery analysis in the reward-seeking task. We recovered the parameter values by fitting the model to the simulated data. For each of the five parameters, the recovered parameter estimates are plotted against the original ones employed in the simulation. Note that values of the *inverse temperature* parameter were log-transformed because of the severe non-normality (skewness > 2 and kurtosis > 7).

(k) Parameter recovery analysis in the loss-avoidance task. Same format as in (j).

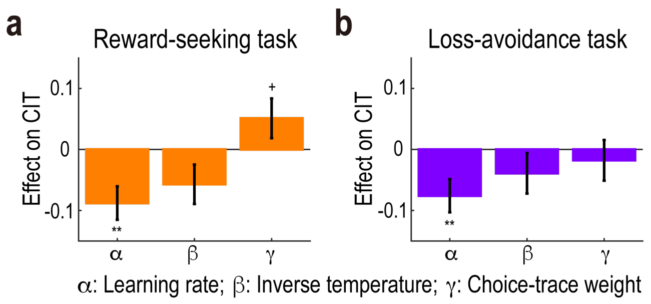


**Fig. S5: Supplementary computational model-based analysis**

(a) Effects of RL parameters on the CIT factor in the reward-seeking task without controlling for the effects of demographic information (mean ± SEM, $N$ = 939). $^{**}P < 0.01$ and $^{+}P < 0.10$; FDR-corrected by the number of tests (i.e., 3) in one-tailed $t$-tests ($\alpha$: corrected $P$ = 0.002 and uncorrected $P$ < 0.001; $\beta$: corrected $P$ = 0.058 and uncorrected $P$ = 0.039; $\gamma$: corrected and uncorrected $Ps$ = 0.058). RL, reinforcement learning; and CIT, 'compulsive behaviour and intrusive thought'. Same format as in Fig. 5cd.

(b) Effects of RL parameters on the CIT factor in the loss-avoidance task without controlling for the effects of demographic information (mean ± SEM, $N$ = 961). Same format as in (a) ($\alpha$: corrected $P$ = 0.008 and uncorrected $P$ = 0.002; $\beta$: corrected $P$ = 0.180 and uncorrected $P$ = 0.120; $\gamma$: corrected and uncorrected $Ps$ = 0.297).

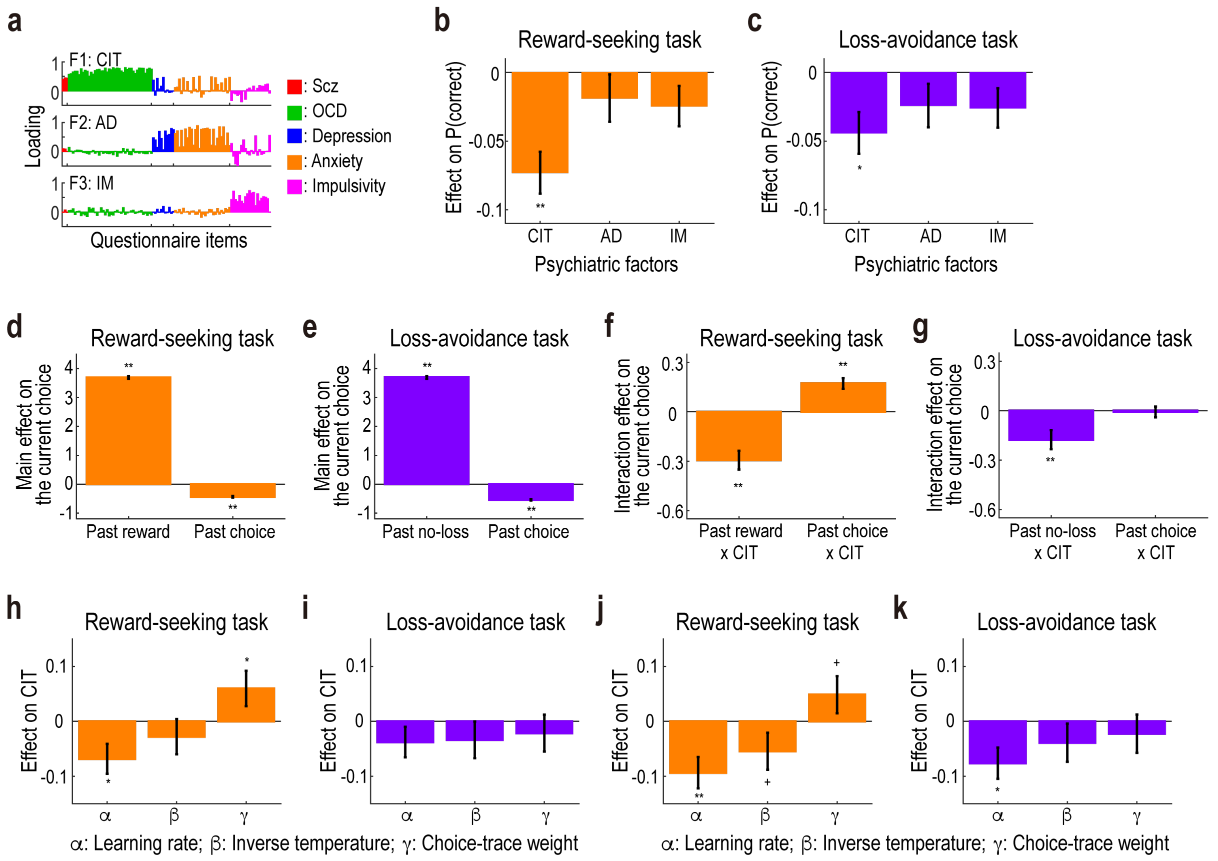


**Fig. S6: Supplementary analysis using data from the reduced questionnaire**

(a) Loadings in the three factors (dimensions) underlying psychiatric symptoms. Loadings of the 104 questionnaire items are shown; 50 of the 154 items were discarded based on the loadings in the original factor analysis (cut-off value = 0.4; see Fig. 2 and Table S3). CIT, compulsive behaviour and intrusive thought; AD, anxiety-depression; and IM, impulsivity.

(b) Effects of psychiatric factors on the proportion of correct choices in the reward-seeking task (mean ± SEM, $n$ = 465,435). Same format as in Fig. 3a. **$P < 0.01$ and *$P < 0.05$, FDR-corrected by the number of tests (i.e., 3) in two-tailed $t$-tests (effect of CIT: corrected and uncorrected $Ps < 0.001$; AD: corrected and uncorrected $Ps = 0.278$; and IM: corrected $P = 0.141$ and uncorrected $P = 0.094$). CIT, compulsive behaviour and intrusive thought; AD, anxiety-depression; and IM, impulsivity.

(c) Effects of psychiatric factors on the proportion of correct choices in the loss-avoidance task (mean ± SEM, $n$ = 476,115). Same format as in Fig. 3b (effect of CIT: corrected $P$ = 0.011 and uncorrected $P$ = 0.004; AD: corrected and uncorrected $Ps$ = 0.129; and IM: corrected $P$ = 0.108 and uncorrected $P$ = 0.072).

(d) Main effects of past rewards and past choices on the current choice in the reward-seeking task (mean $\pm$ SEM, $n$ = 461,679). Same format as in Fig. 4a. **$P < 0.01$, FDR-corrected by the number of tests (i.e., 2) in two-tailed $t$-test (Past reward: corrected and uncorrected $Ps < 0.001$; Past choice: corrected and uncorrected $Ps < 0.001$).

(e) Main effects of past no-losses and past choices on the current choice in the loss-avoidance task (mean $\pm$ SEM, $n$ = 472,271). Same format as in Fig. 4b (Past no-loss: corrected and uncorrected $Ps < 0.001$; Past choice: corrected and uncorrected $Ps < 0.001$).

(f) Interaction effects on the current choice in the reward-seeking task (mean $\pm$ SEM, $n$ = 461,679). Same format as in Fig. 4c. **$P < 0.01$, FDR-corrected by the number of tests (i.e., 2) in two-tailed $t$-tests (Past reward x CIT: corrected and uncorrected $Ps < 0.001$; Past choice x CIT: corrected and uncorrected $Ps < 0.001$). CIT, compulsive behaviour and intrusive thought.

(g) Interaction effects on the current choice in the loss-avoidance task (mean $\pm$ SEM, $n$ = 472,271). Same format as in Fig. 4d (Past no-loss x CIT: corrected $P = 0.005$ and uncorrected $P = 0.002$; Past choice x CIT: corrected and uncorrected $Ps = 0.808$).

(h) Effects of RL parameters on the CIT factor in the reward-seeking task (mean $\pm$ SEM, $N$ = 939). *$P < 0.05$; FDR-corrected by the number of tests (i.e., 3) in one-tailed $t$-tests ($\alpha$: corrected $P = 0.019$ and uncorrected $P = 0.006$; $\beta$: corrected and uncorrected $Ps = 0.193$; $\gamma$: corrected $P = 0.047$ and uncorrected $P = 0.031$). RL, reinforcement learning; and CIT, compulsive behaviour and intrusive thought. Same format as in Fig. 5cd.

(i) Effects of RL parameters on the CIT factor in the loss-avoidance task (mean $\pm$ SEM, $N$ = 961). Same format as in (h) ($\alpha$: corrected $P = 0.235$ and uncorrected $P = 0.088$; $\beta$: corrected $P = 0.235$ and uncorrected $P = 0.156$; $\gamma$: corrected and uncorrected $Ps = 0.261$).

(j) Effects of RL parameters on the CIT factor in the reward-seeking task without controlling for the effects of demographic information (mean $\pm$ SEM, $N$ = 939). Same format as in (h). **$P < 0.01$ and $^{+}P < 0.10$; FDR-corrected by the number of tests (i.e., 3) in one-tailed $t$-tests ($\alpha$: corrected $P = 0.001$ and uncorrected $P < 0.001$; $\beta$: corrected $P = 0.076$ and uncorrected $P = 0.053$; $\gamma$: corrected and uncorrected $Ps = 0.076$).

(k) Effects of RL parameters on the CIT factor in the loss-avoidance task without controlling for the effects of demographic information (mean $\pm$ SEM, $N$ = 961). Same format as in

(h) ($\alpha$: corrected *P* = 0.011 and uncorrected *P* = 0.004; $\beta$: corrected *P* = 0.196 and uncorrected *P* = 0.131; $\gamma$: corrected and uncorrected *Ps* = 0.259).

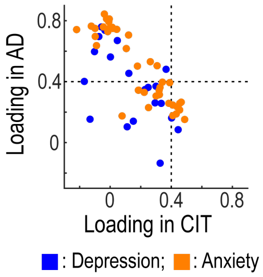


**Fig. S7: Supplementary factor analysis of questionnaire data**

Loadings in the CIT (compulsive behaviour and intrusive thought) and the AD (anxiety-depression) factors. *Blue* and *orange* points denote the questionnaire items related to depression and anxiety, respectively. Dashed lines indicate a conventional cut-off value of loading: 0.4.
